# Lowered Abundance of Gut Bacteriophage Species Is Associated With Human Cancer Cachexia

**DOI:** 10.1002/jcsm.70324

**Published:** 2026-06-07

**Authors:** Axel Stang, Thomas Illig, Karsten Hiller, Hauke Weilert, Rudi Schmidt, Raphael Gronauer, Martin Seifert

**Affiliations:** ^1^ Department of Haematology, Oncology and Palliative Care Medicine Asklepios Hospital Barmbek Hamburg Germany; ^2^ Asklepios Tumorzentrum Hamburg Hamburg Germany; ^3^ Asklepios Medical School Hamburg Germany; ^4^ Asklepios Campus Hamburg Semmelweis University Budapest Hungary; ^5^ Department of Human Genetics Hannover Medical School Hannover Germany; ^6^ Hannover Unified Biobank (HUB) Hannover Germany; ^7^ Department of Bioinformatics and Biochemistry, Braunschweig Integrated Centre of Systems Biology (BRICS) Technische Universität Braunschweig Braunschweig Germany; ^8^ Immunetrue Cologne Germany; ^9^ Department of Bioinformatics Universität Innsbruck Innsbruck Austria; ^10^ 10x Genomics Leiden the Netherlands

**Keywords:** cancer cachexia, gut bacteriophages, gut microbiome, metagenomics

## Abstract

**Background:**

Cancer cachexia exemplifies a high medical need condition without effective treatment. Recent studies implicated bacterial gut microbiome alterations to cancer cachexia. Whether the gut bacteriophage profile, an important microbiome component for health and disease, is also related to cancer cachexia remains unknown. We aimed to profile gut microbiome alterations in human cancer cachexia with attention on bacteriophages.

**Methods:**

We performed shotgun metagenomic sequencing in stool samples from 78 cachectic and 42 noncachectic patients (53% male, mean age 67 ± 8 years) with newly diagnosed, advanced‐stage (UICC IV) gastrointestinal cancers. Cachexia was defined according to the main criterion agreed upon international consensus (weight loss [WL] adjusted to body mass index [BMI]). Obtained DNA short‐reads were used for *k*‐mers‐based, phage‐inclusive matching with reference databases, de novo phage assembly and inferring microbiome‐encoded functions. We replicated significance‐based statistical and prediction‐oriented machine‐learning analyses in 2022 and 2025 generated metagenome datasets to incorporate the recent change by the International Committee on Taxonomy of Viruses (ICTV) from morphology‐based (valid until 2022) to revised genome‐based phage taxonomy into microbiome findings of cachexia.

**Results:**

Cachectic and noncachectic patients differed significantly regarding BMI (mean 20.9 vs. 26.4 kg/m2), WL (mean −6.5 vs. −0.2 kg), survival (median 5 vs. 13 months) and clinical cachexia domains (e.g., C‐reactive proteine and appetite loss) (all *p* < 0.001) but not for other clinical covariables (e.g., cancer type) (all *p* > 0.05). Read‐based mapping (2022/2025) identified 1.312/1.513 species (74/39 phage species), and de novo assembly resulted in 4.184/4.209 contigs (corresponding to 65/39 phage species). Concordantly, both analyses (2022 and 2025) showed that prevalent cachexia associated significantly with beta‐diversity (Bray‐Curtis distance, PERMANOVA, *p* < 0.05), but not to alpha‐diversity (Shannon‐Index, ANOVA, *p* > 0.05), reduced microbiome‐encoded detoxification functions (e.g., enriched microbial β‐glucuronidase and depleted bacterial efflux pumps) and lowered abundance of bacterial species with false‐discovery‐rate (FDR)‐corrected *p <* 0.05 (2022: *Faecalibacterium prausnitzii* , *Roseburia intestinalis* , *Streptococcus* species and *Lachnospiraceae* species; 2025: *Faecalibacterium* species, *Ruminococcus gauvreauii* and *Intestinibacter bartlettii*). Further, lowered abundance of bacteriophages associated with cachexia, predominantly affecting double‐stranded (2022: *Caudovirales*, *Siphoviridae*, FDR‐corrected *p* < 0.05; 2025: *Myoviridae*, *Siphoridae*, *p* < 0.05) but also single‐stranded (2022: *Inoviridae*, *Microviridae*, *p* < 0.05; 2025: *Inoviridae*; *p* < 0.05) DNA phage species. In machine‐learning models, bacteriophages were top‐ranked cachexia predictors (2022: *Caudovirales*, *Siphoviridae*; 2025: *Myoviridae*, *Siphoridae*). Accuracy was highest when only phage contigs were taken into account (correctly classified instances: 75.0%–85.8%; AUC: 0.703–0.916).

**Conclusions:**

The previously unknown link between gut bacteriophages and human cancer cachexia expands the scope for basic, translational and clinical microbiome‐targeted research in an area of significant unmet medical need.

**Trial Registration:**

Study Box of the German Cancer Society (Registration Number ST‐U069, Date: 29 May 2018)

## 1 Introduction

Cancer cachexia is a complex body‐wasting syndrome clinically featured by unintended weight loss (WL), affecting up to 80% of patients at diagnosis of advanced‐stage cancer, particularly those with gastrointestinal cancers [1, 2]. The syndrome compromises patients' well‐being, prognosis and treatment outcomes and accounts for 20%–30% of cancer‐related deaths [3, 4]. Despite some promising early clinical data for ponsegromab, a novel inhibitor of the circulating cytokine GDF‐15 [5], there are currently no approved medications for treating cancer cachexia [S1, S2]. Recent studies, mostly based on 16S rRNA gene amplicon sequencing, implicated alterations of the bacterial gut microbiome composition to cancer cachexia [6, 7, 8, 9, 10]. Whether the gut viral community, predominantly composed of DNA phages (mainly double‐stranded DNA [dsDNA] but also single‐stranded DNA [ssDNA] phages [11]) is also altered in cancer cachexia is still unknown. The biomedical relevance of the complex community of gut‐residing phages (phageome) gains increasing attention in medical research, because changes of the gut phageome composition can influence bacterial populations, metabolic processes and immune responses with implications for the development of disease [12], [S3]. However, investigation of the gut phageome is a relatively novel field, with new gene sequences being published at an increasing rate, and a large percentage of unknown sequences, termed ‘gut viral dark matter’ [11, 12], [S4].

Bacteriophages are viruses that infect bacteria and can shape bacterial populations through a variety of mechanisms: by lysing their bacterial targets (lytic phages), integrating as prophages into the bacterial genome (temperate phages, lysogeny), by staying in an extrachromosomal state (plasmid phages and pseudolysogeny) and/or by transferring bacterial genes (phage transduction), which can confer advantages to the bacterial host (e.g., antibiotic resistance) [13], [S5]. Although these phage‐bacteria interactions are important to maintaining gut and overall health [14], [S5], the composition of regular gut phage constituents is altered in various diseases, including metabolic conditions, such as obesity, diabetes and stunted children [S7–S10]. Functionally, phage‐encoded genes influence the bacterial host metabolism, such as vitamin, amino acid and short‐chain fatty acid synthesis, which impacts human host metabolism, as 15% of the metabolites in human blood originate from the gut microbiome [S11]. At the human host‐gut interface, gut phages can pass through the epithelial layer and activate immune and/or inflammatory processes in the mucosa, with implications for systemic immune reactions [S12, S13]. Cross‐disease analyses converge on two mechanisms affecting the immuno‐metabolic cross‐talk: a reduced abundance of gut phages with loss‐of‐function for beneficial effects or the expansion of gut phages with gain‐of‐functions with pathogenic potential [S14]. However, establishing causality beyond correlation in the complex interplay between gut phages, bacteria, human host and disease pathogenesis, especially in tumour‐bearing hosts with an additional factor at play, is difficult. Challenges also arise from the recent International Committee on Taxonomy of Viruses (ICTV) revision. In 2022, the ICTV significantly changed the phage taxonomy and nomenclature from a morphology‐based to a genome‐based classification, which newly created, promoted, renamed or removed phage orders, families, subfamilies, genera and species; the most important change was the abolishment of the large morphological family *Siphoviridae* and order *Caudovirales* [15, 16]. At present, however, no standardized phage classification workflow for gut phage identification and associations with disease exists, and the impact on reliability, reproducibility, conclusions, and cross‐trial comparability of research data under different ICTV frameworks remains unclear [17, 18, 19].

Shotgun metagenomic sequencing of stool samples enables the study of genomic DNA from all gut microorganisms; however, phage‐inclusive microbiome profiling poses challenges. First, < 6% of total microbial DNA in stool samples represents phage DNA (93% is bacterial DNA), making it generally difficult to identify low‐abundant phage sequences in metagenomics datasets [11]. Second, read‐based reference‐dependant identification of gut DNA phages is limited by incomplete phage genome databases [12], [S4]. Methods to improve phage identification are *k*‐mer‐based software tools utilizing all possible sequences of length k from DNA reads, thereby increasing the reads per sample and the fraction of reads assignable to phage genomes in databases [20, 21], and/or to assemble sequencing reads into contigs and identify phage genomes de novo [22, 23]. Third, recent ICTV taxonomy updates significantly changed phage taxonomy and nomenclature, yet a standardized phage classification workflow does not exist [17, 18, 19]. Fourth, differential abundance analyses of compositional microbiome data with many features but limited sample size require false discovery control by complementary methods that differentially process the data and validate each other [24], [S15]. Shared findings from both significance‐based statistical (direct species‐level testing between sample groups) and prediction‐oriented machine‐learning (ML) methods (train models to label groups of samples) lend strengths for capturing truly different taxon‐based abundances between sample sets or patient groups [25].

In this study, we compare shotgun metagenomic sequencing data of total DNA from stool samples of cancer patients with and without cachexia. We matched the obtained DNA short‐reads based on *k*‐mers with reference databases, generated de novo viral contigs, applied different statistical approaches to determine overlapping significant cachexia‐related taxon‐based alterations and assessed the performance of top taxa predictors of microbiome‐based ML models for prediction of cachexia. The aim of the study was to identify gut microbiome alterations in human cancer cachexia with attention on bacteriophages. We replicated our analyses in metagenomic datasets generated in 2022 and 2025 to take account for the potential impact of the recent change from the morphology‐based to the revised genome‐based ICTV phage taxonomy.

## 2 Methods

### 2.1 Study Population

For this single‐centre cross‐sectional case–control study, clinic‐based recruitment was targeted to patients with newly diagnosed metastatic cancer presenting at the oncology units of the Asklepios Hospital Barmbek, between June 2018 and August 2021. Eligible patients met the following inclusion criteria: age ≥ 18 years, pathology‐confirmed metastatic disease from gastric, colorectal, pancreatic, liver or ovarian cancer and no anticancer treatment before study inclusion. Exclusion criteria included acute or chronic diarrhoea, acute gastrointestinal illness including ileus, inflammatory bowel disease, antibiotic medication past 3 months, autoimmune diseases, immunosuppressive therapy including corticosteroids, acquired immunodeficiency syndrome, kidney or liver failure and need for emergency surgery.

### 2.2 Clinical Assessments

Data collection consisted of one part to be completed by the study participants and one to be completed by the study coordinators. A research assistant was available and provided help in a face‐to‐face interview as necessary. Clinical data were collected from medical records by the study coordinators at the time of study inclusion and during follow‐up (date of death). Performance status was recorded as Eastern Cooperative Oncology Group (ECOG) PS [S16]. Patient‐reported data on height, body weight, WL history, body mass index (BMI), appetite, food intake, vegetarian diet, smoking and alcohol consumption were collected by means of a structured questionnaire. Information about actual height and weight, WL at last 6 months, and food intake past 6 months (unchanged or reduced) was provided by the patients using questions from the Scored Patient‐Generated Subjective Global Assessment (PG‐SGA) [S17]. Assessment of appetite was performed using a numerical rating scale provided by the Edmonton Symptom Assessment System (ESAS) [S18]. Diet‐based vegetarianism was determined from the intake of animal products (red meat, poultry, fish, dairy products and eggs). Vegetarians were defined by a plant‐based dietary pattern that excludes red meat and, to different extents, other animal products (subtypes ranged from pesco‐lacto‐ovo‐vegetarians to vegans). Smoking was defined as current daily smoking (including cigars, pipes or electronic cigarettes). Self‐reported alcohol consumption using Questions 1–3 of the Alcohol Use Disorder Identification Test (AUDIT) was converted into grammes per week (1 standard drink equivalent to 14 g of alcohol) [S19]. Prevalent diabetes was defined as self‐reported diabetes, diabetes diagnosis in medical records and/or use of diabetes medications. Prevalent heart disease was assessed as a history of hospitalization due to myocardial infarction, coronary disease (includes heart attack and angina pectoris chest pain), heart failure and/or cardiac dysrhythmia. Medication use was defined as drug intake during the last 3 months. Cachexia was defined based on BMI‐adjusted WL in the past 6 months using the criteria from the international consensus described by Fearon et al. [1]. Patients were classified as cachectic who had (1) involuntary WL > 5% over the past 6 months or (2) BMI < 20 kg/m^2^ and involuntary WL > 2% over the last 6 months. Patients were classified as noncachectic if they experienced no or ≤ 5% (≤ 2% for BMI < 20 kg/m^2^) involuntary WL over the last 6 months.

### 2.3 Stool Sampling and Storage

Stool samples were collected using a stool catcher to avoid toilet contamination. Using a sterile spoon, two pieces of stool (cherry stone size, ~3–5 g of faeces) were transferred into a sterile plastic tube filled with 3.5‐mL RNA stabilizing solution (Biosepar, Simbach, Austria), suspended with the integrated screw cap stirrer until obtaining a homogeneous faecal sample mixture and refrigerated at 4°C in a portable cooler with ice packs. Samples were then stored within ≤ 12 h at −80°C for an average of 1.2 ± 0.6 years until further processing.

### 2.4 Stool DNA Extraction and Shotgun Metagenomic Sequencing

Metagenomics sequencing including DNA extraction from stool samples was performed applying the metagenome sequencing service from Eurofins Genomics (Konstanz, Germany). In brief, stool samples were thawed on ice, aliquoted (1.000 μL), and then DNA was extracted with the DNeasy PowerSoil Pro Kit (Qiagen, Hilden, Germany) following the manufacturer's protocol. For enzymatic DNA fragmentation, DNA was quantified for each sample and diluted to a concentration of 0.3 ng/μL. The sequencing library was built from a total DNA input of 1.5 ng, which was normalized to a volume of 5 μL of DNA at 0.3 ng/μL, and was prepared using the NEB‐Kit NEBNext Ultra II FS DNA Library Prep Kit for Illumina. Next, the prepared libraries were sequenced on the Illumina NovaSeq 6000 system (Illumina, San Diego, California, United States) with a run configuration of 2x150 bp paired‐end mode (sequencing depth: > 10 Gb, ≥ 10 million read pairs and ≥ 20 million raw reads per sample) at Eurofins (Konstanz, Germany). Eurofins offers a rigorous sample processing workflow termed INVIEW metagenome program (all‐in‐one in‐house solution), which is designed to detect and remove host DNA, contaminant sequences, sequence artefacts and low‐quality reads that do not meet strict quality control (QC) scores using established guidelines, standards and bioinformatic quality control (QC) tools for quality assurance (QA) of shotgun metagenomic data from stool samples [S20]. In brief, steril water was used as negative control to detect contamination and underwent all steps of sample preparation, DNA extraction, library preparation and sequencing in parallel with the biological samples. Cross‐sectional and longitudinal run‐/and sample‐level QC metrics (e.g., to detect contaminant sequences) were monitored through the Sequencing Analysis Viewer (Illumina; https://illumina.github.io/interop/indexhtml). The raw data files produced by Illumina were de‐multiplexed in BaseSpaceTM (Illumina computing platform) and converted into FASTA files (Illumina BCL Convert v3.9.3). These raw reads were subject to trimming and filtering using the QC tools FastQC (v0.11.9), MultiQC (v1.12) and Trimmomatic (v0.39); human reads were mapped and filtered using the human genome reference GRCh38 (p12) and Bowtie2 (v2.4.2). The most important default settings for quality value thresholds for removing low‐quality raw reads were as follows: (1) the Phred score, which measures the nucleotide base‐call error probality (low‐quality reads: do not contain ≥ 75 Q30 bases among the first 35 cycles or < Q20 bases after trimming [Q30 and Q20 define a base‐call error probability of 0.001 and 0.01, respectively]); (2) the GC distribution and content, which identifies systematic per‐sequence biases, sample contamination and library preparation issues (low‐quality sequences: do show GC peaks off‐centre and do not show a Gaussian GC curve distribution with average GC content between 40% and 60%); (3) the adapter content, which measures technical sequence artefacts inserted by the sequencing machine which are not part of the biological sample (low quality samples: > 10% of the reads contain adapters); and (4) read lengths < 50 bp after adapter trimming and quality filtering. In FastQC and MultiQC reports after trimming, all samples passed the QC checks (adapter content > 5% but < 10% for 5 of 120 samples [4.1%], only warning issued, no failure). FastQC per‐base sequence content plots showed *K*‐mer bias for ‘GGGGGGG’ for all samples, which is due and normal for the random prime based library. The QC‐checked number of high‐quality reads per sample ranged between 19.6 and 34 million reads per sample.

### 2.5 De Novo Assembly of Viral Contigs

The software Metaviral‐SPAdes (2022: v3.14.1; 2025: v3.15.3, accessed through usegalaxy.org) [22] was used for de novo assembly of metagenomics contigs for each of the 120 metagenomes. MetaviralSPAdes is based on a set of virus‐specific Hidden Markov Models (HMMs) derived from viral genomes from the NBCI reference (RefSeq) nucleotide (NT) and PFAM‐A database (v31). The obtained contigs were then mapped against the NCBI RefSeq NT database (2022: r212; 2025: r229) using BLASTn (2022: v2.12.0; 2025: v2.17.0) to annotate the sequences. To get a semiquantitative measure of the abundance of *de novo* assembled bacteriophage sequences, sequences of all de novo generated contigs derived from Metaviral‐SPAdes were combined. Next, Burrows–Wheeler Alignment (BWA) tool (2022: v0.7.17; 2025: v0.7.19, accessed through github.com/ih3/bwa) was used to build a mapping index. Metagenomics sequences of each sample were then mapped to the assembly‐based sequencing library using BWA‐MEM functionality. After mapping, feature count was used to count reads mapped to different contigs and normalized using counts per million (CPM). To assess life cycle assignment of the obtained contigs, the hybrid machine learning and protein similarity software VIBRANT (v1.2.1; accessed through https://github.com/AnantharamanLab/VIBRANT) [23] was applied.

### 2.6 Taxonomy and Abundance Estimation

The *k*‐mer‐based sequence mapper kraken2 (v2.1.2; accessed github.com/Derrick/Wood/kraken2) [20, 21] was applied to assign metagenomic sequencing reads to taxonomic labels in mapping libraries with a confidence score of 0.6 for sequence mappings to increase stringency (default setting: confidence score of 0.0). A default option of kraken2 was used to build a kraken2 database using the NBCI BLAST NT database. The data download (https://genome‐idx.s3.amazonas.com/kraken/k2_core_nt20241228.tar.gz) was also the basis for the blast nucleotide search (https://ftp.ncbi.nlm.nih.gov/blast/db/FASTA/nt.gz). The data were downloaded with the command ‘kraken2‐built—download‐library nt’ on 22 October 2022 and on 18 March 2025 to build a library on the NT‐database (2022: r212.0; 2025: r229) with a *k*‐mer size of 40 nucleotides. All reads were assigned to the taxon with the highest total hits of *k*‐mers matched by pruning the general taxonomic trees affiliated with mapped sequences. Relative taxonomic abundance was calculated using the Bracken (Bayesian re‐estimation of abundance with Kraken) algorithm, which is a highly accurate statistical method that computes the abundance of species in DNA sequences from a metagenomics sample [S21]. Kraken's original taxonomic classification assignments underwent probabilistic reassignment by Bracken (2022: v0.7.17; 2025: v0.7.19) using the command ‘bracken‐build’ to establish the according libraries for the 150‐bp read length.

### 2.7 Functional Abundance Estimation

Functional microbiome‐encoded annotation was based on the integrated gene catalogue (IGC, v1/9.9 M) comprising 9.879.896 nonredundant reference genes in the human gut microbiome [S22]. Obtained DNA sequencing data were mapped with BWA‐MEM function (v0.7.17, accessed through github.com/ih3/bwa) against the IGC Human Gut Reference Catalogue (v1/9.9 M) downloaded from the China National Gene Bank repository (https://db.cngb.org/microbiome/genecatalog/genecatalog_human/). The annotation provided by the IGC consortium was used to assign genes to functional categories using the KEGG Orthology (KO) database (r104). Normalized data (counts per million with a > 100 reads cut‐off) were used for KO grouping and mapping to KEGG pathways.

### 2.8 Statistical Methods

Group‐wise comparisons of cachectic versus noncachectic cancer patients with regards to items representing cachexia domains and clinical data were performed. Continuous variables with normal distribution are presented as mean (standard deviation) and compared using Welch's two sample *t* test. Continuous variables outside the normal distribution are presented as medians (quartiles 1 and 3) and compared using Wilcoxon rank sum test. Categorical variables are summarized as counts (percentages) and compared using Pearson's Chi‐squared test. The relationship between cancer type and gut microbial composition was additionally assessed by hierarchical clustering of samples using the complete linkage method with Euclidean distance as distance metrics. Overall survival (OS) was estimated using Kaplan–Meyer methods and compared using the log rank test. Time was censored at the time of death or at the date of last follow‐up for patients being alive. For all tests, *p* values < 0.05 (two‐sided) were considered statistically significant (R software, v4.1.1). Alpha and beta microbiome diversity was calculated with R package vegan (v2.5‐7) and plotted with the ggplot2 package (r3.3.6 and r4.0.1). ANOVA was used to determine significant differences for α‐diversity (indicated by Shannon index) and PERMANOVA with 999 random permutations to assess significant differences for β‐diversity (indicated by Bray‐Curtis distances) between groups. Differential abundance analysis was based on two‐tailed unpaired *t* test (pairwise comparison of nontransformed mean abundance values) and LinDa analysis (linear regression of log2‐transformed data). To limit false‐positive findings, we applied Benjamini‐Hochberg false discovery correction (FDR) and defined overlapping significance of FDR‐corrected *p* values < 0.05 in both *t* test and LinDA analysis as the general cut‐off for statistically different abundances between groups. To still attend tendencies, especially for very low‐abundant species below ≤ 0.1% relative mean basic abundance in group‐wise comparison, we separately performed a correlation analysis for species that referred to the nominal *p* values of < 0.05 in *t* test and/or LinDa analysis.

### 2.9 Machine Learning Classification

To account for combinatorial effects and interrelations between taxa in our high‐dimensional dataset (number of features larger than sample size), we established random forest (RF) models to develop a predictive ML model for binominal classification between cachectic and noncachectic patients and to generate measures of feature importance. The Waikato Environment for Knowledge Analysis (Weka, v3.8.6) (https://www.cs.waikato.ac.nz/ml/weka/) was used as workbench [25]. The RF approaches run either on taxa input features inferred by the NT‐database or taxa input features obtained by de novo assembly. Prior to RF model training, a feature selection was performed, and only species that showed nominally significant differential abundance in *t*‐test and/or LinDA analysis (*p* < 0.05) were used as features for training. To address the imbalance between the number of cachectic and noncachectic patients, we applied a meta‐classifier approach with reweighted training instances, making the base RF classifier cost‐sensitive to balance false positives and false negatives, reduce false discoveries and improve overall predictive accuracy. For validation, we used data‐split and tenfold cross‐validation, ensuring each fold contained a balanced proportion of both groups to handle dataset imbalances and reduce overfitting. After applying the trained RF model to classify the left‐out test set, RF model's predictive performance was assessed using receiver operating characteristic (ROC) methods. ROC curves were computed as the mean across the tenfolds, and the corresponding area under the curve (AUC) was calculated. Additionally, we assessed precision, recall, F‐measure and true and false‐positive rates. Feature importance in the RF model was assessed using the metrics mean decrease in impurity (Gini importance) and the number of nodes in which the predictor appeared.

## 3 Results

### 3.1 Baseline Characteristics of the Study Population

**FIGURE 1:**
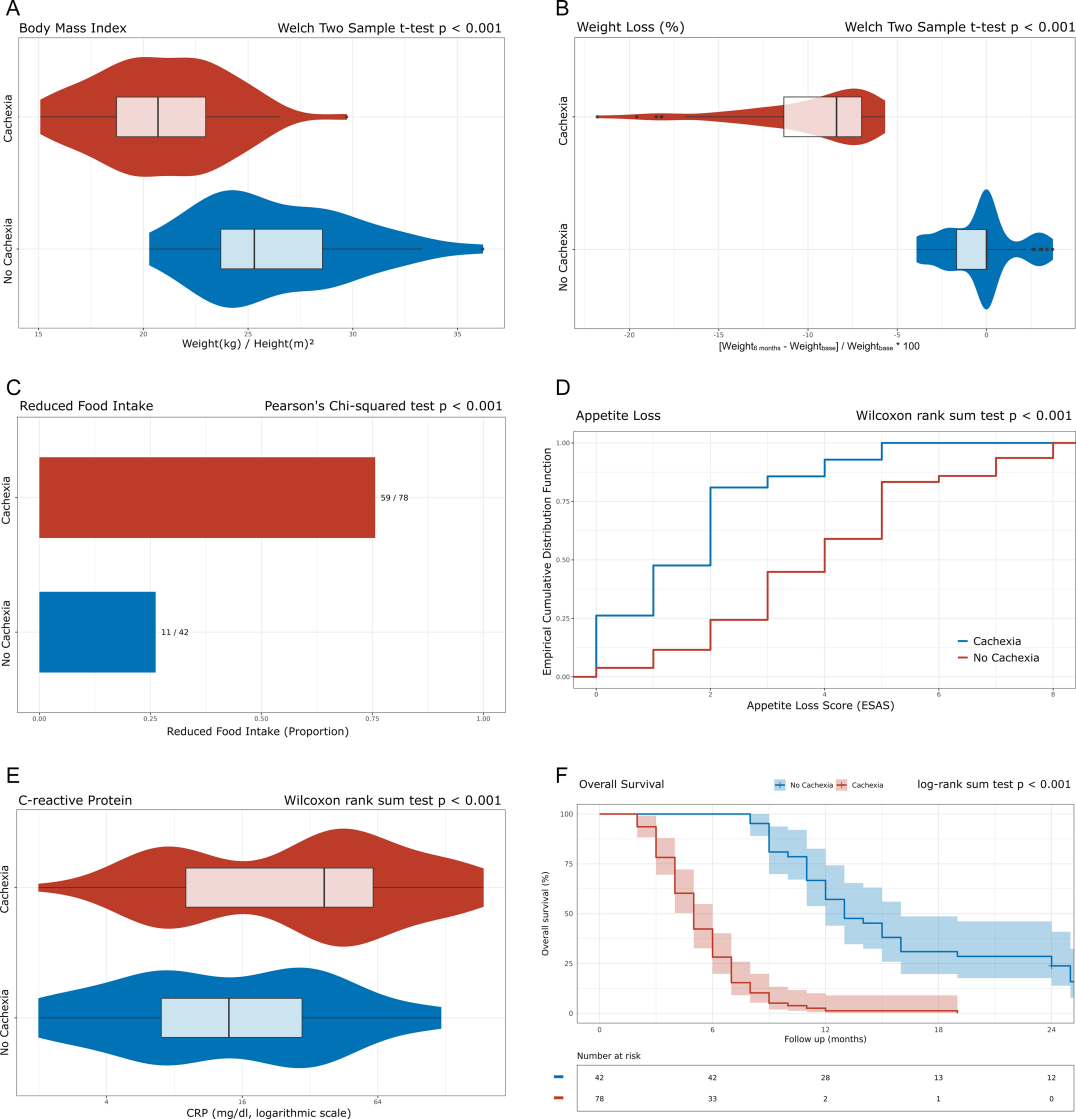
Significant differences of cachexia domains and survival between cachectic (*n* = 78) and noncachectic (*n* = 42) cancer patients. (A) Body mass index (BMI) difference (Welch two sample *t* test: *p* < 0.001). (B) Percent weight loss (WL) difference past 6 months (Welch two sample *t* test: *p* < 0.001). (C) Food intake difference (Pearson's chi‐squared test, *p* < 0.001). (D) Appetite loss score difference (Wilcoxon rank sum test, *p* < 0.001). (E) C‐reactive (CRP) blood level difference (Wilcoxon rank sum test, *p* < 0.001). (F) Overall survival (OS) difference (log‐rank test, *p* < 0.001). Violin plots in Figure 1A,B,E show the median (middle line), 25th and 75th percentile (box), and the line surrounding the box plots (Kernel density estimation boundary) represents the frequency of data points at a given value, with wider sections indicating higher density and narrower sections indicating lower density. The shadowed bands around the solid Kaplan–Meier curve lines in Figure 1F represent the 95% confidence interval of estimated OS probabilities over time.

**FIGURE 2:**
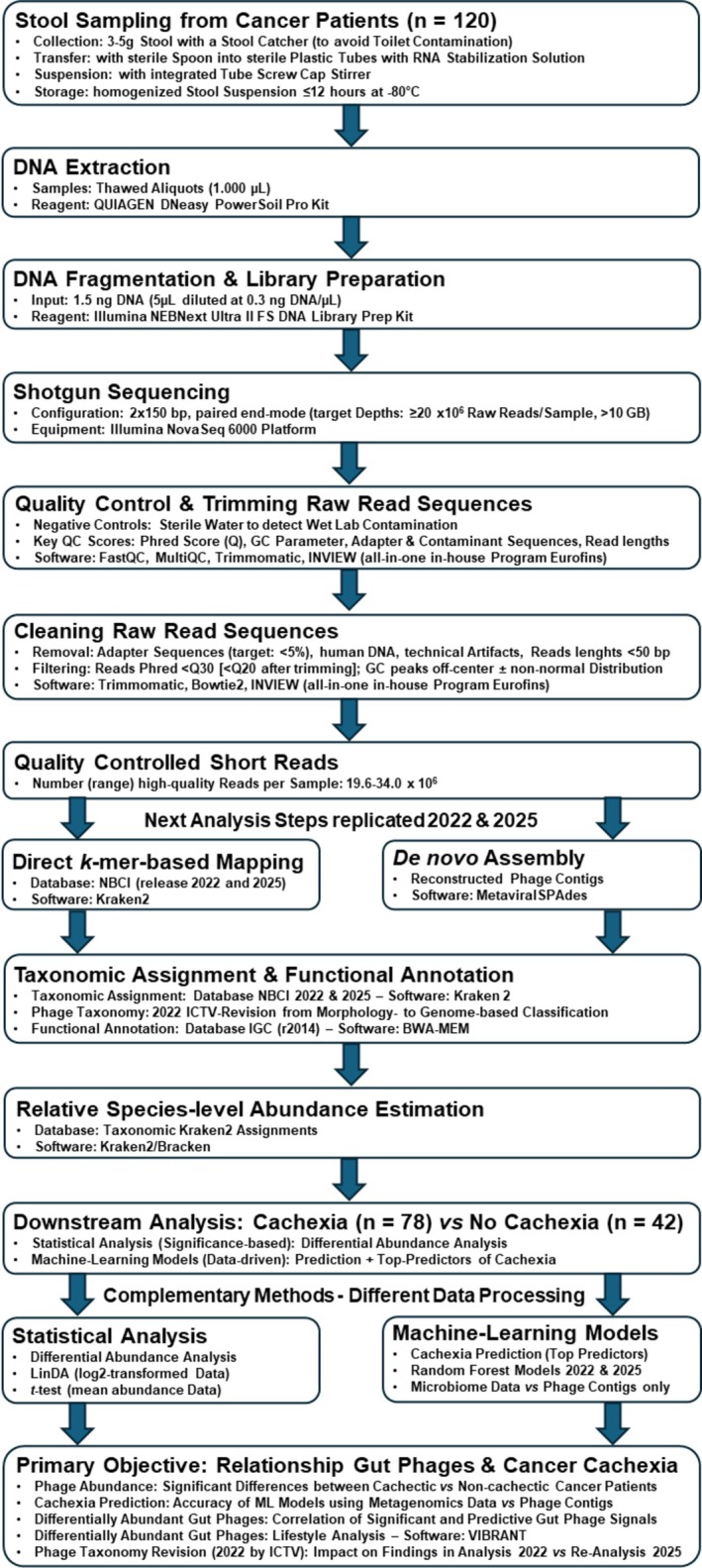
Flowchart summarizing the key steps, methods, workflow and main focus of the analysis procedures for the phage‐inclusive shotgun metagenomic profiling from stool samples of cancer patients (*n* = 120). Note that the taxonomic annotation and abundance estimation of short‐reads (direct *k*‐mer‐based matching) and de novo assembled phage contigs, as well as the comparative downstream analyses between cachectic (*n* = 78) and noncachectic (*n* = 42) cancer patients, were replicated in 2022 and 2025 to incorporate the change by the International Committee on Taxonomy of Viruses (ICTV) in 2022 from morphology‐based to revised genome‐based phage taxonomy into the gut microbiome findings of cachexia. GC, guanin cytosine; ICG, integrated gene catalogue; NBCI, National Center for Biotechnology Information (US); QC, quality control.

In total, 120 cancer patients with metastatic disease (stage UICC IV) participated in the study. Forty‐one patients were diagnosed with colorectal cancer, 32 with pancreatic cancer, 30 with gastric cancer, 12 with liver cancer and 5 with peritoneal carcinosis from ovarian cancer. Among these, 78 patients were classified as cachectic, whereas 42 patients were noncachectic (Table 1). The cachectic patients had a mean BMI of 20.9 kg/m^2^ and a mean WL of 6.5 kg (mean %WL: −9.7%), whereas the noncachectic patients had a mean BMI of 26.4 kg/m^2^ without WL (Table 1 and Figure 1A,B; *p* < 0.001, respectively). Both groups had almost similar within‐group distribution and no significant between‐group difference of clinical covariables, including an almost similar distribution pattern with regards to cancer types (Table 1, *p* > 0.05, respectively). By contrast, both groups were significantly distinct with regards to key cachexia domains (CRP level, appetite loss, food intake, ECOG‐PS; Figure 1C–E; *p* < 0.001, respectively). Further, after a median follow‐up time (last update: 31.12.2023) of 35 months (interquartile [IQR] range 30–49 months), the median OS (all patients: 7 months [95% confidence interval 6–8 months]) was significantly shorter for cachectic compared to noncachectic patients (5 vs. 13 months; Figure 1F, *p* < 0.001). The metagenomics datasets collected from 78 cachectic and 42 noncachectic patients were compared for taxa inferred by the NT‐database. In 2022, Kraken2 *k*‐mer‐based mapping identified 1.312 species, from which 74 were phage or viral species, and in 2025 1.513 species, from which 39 were phage or viral species. Metagenomic de novo assembly resulted 2022 in 4.184 assembled contigs, corresponding to 65 phage or viral species, and resulted 2025 in 4.209 assembled contigs, corresponding to 39 phage or viral species. Hierarchical clustering of samples identified no association between the abundance of certain species with the cancer type, neither for the entire cohort, cachexia cohort and/or noncachexia cachexia in the first analysis in 2022 (Figure S1A–C) nor in the re‐analysis in 2025 (Figures S1D–S1F). The workflow for the phage‐inclusive metagenomic profiling of the stool samples from the cancer patients (*n* = 120), including (Step 1) wet laboratory procedures (probe collection, processing, DNA extraction, library preparation, sequencing, quality control, trimming and discard of low‐quality short‐reads), (Step 2) bioinformatics analyses of high‐quality read sequences (taxonomic annotation, de novo assembly of phage contigs and taxa abundance estimation), and (Step 3) comparative statistical and ML‐based analyses between cachectic (*n* = 78) and noncachectic (*n* = 42) cancer patients are summarized in Figure 2. Notably, Step 2 (bioinformatics analysis of short reads) and Step 3 (downstream analysis of cachexia vs. no cachexia) were conducted, respectively, in parallel using complementary methods that process the data differentially in order to identify shared findings that validate each other. Furthermore, Steps 2 and 3 were replicated in an analysis in 2022 and 2025 to incorporate the recent ICTV revision from morphology‐based to genome‐based phage taxonomy and nomenclature into gut microbiome findings of cancer patients exhibiting a cachectic phenotype (Figures 1 and 2).

**TABLE 1:** Demographic, clinical and cachexia domain characteristics of the study population (*n* = 120 cancer patients at diagnosis of advanced‐stage, UICC IV gastrointestinal cancers).

Variable	Cachexia	No cachexia	*p* value
Sample size (*n*)	78	42	
Clinical characteristics			
Demographic data			
Age,^a^ year	68 (10)	65 (12)	0.074
Male^c^	46 (59)	17 (40)	0.053
Life style			
Smoking^c^	23 (29)	10 (24)	0.682
Alcohol^c^	26 (33)	17 (40)	0.713
Vegetarian^c^	5 (6.4)	2 (4.7)	1.0
Comorbidity			
Diabetes	17 (21)	7 (17)	0.644
Heart Disease	6 (7.7)	4 (9.5)	0.729
Cancer type			
Colon cancer^c^	25 (32)	16 (38)	0.707
Pancreatic cancer^c^	23 (29)	9 (21)	0.532
Gastric cancer^c^	20 (26)	10 (24)	1.0
Liver cancer^c^	7 (9.0)	5 (12)	0.754
Ovarian cancer^c^	3 (3.8)	2 (4.8)	1.0
Medication			
Morphine^c^	20 (25)	7 (17)	0.499
Novaminsulfon^c^	23 (29)	9 (21)	0.523
Nonsteroidal analgetics^c^	14 (18)	10 (24)	0.642
Pantozol^c^	8 (10)	7 (16)	0.403
Diuretics^c^	7 (9.0)	5 (12)	0.754
Antibiotics (within ≤ 2 weeks)^c^	20 (25)	10 (24)	1.0
No antibiotics (within ≥ 3 months)^c^	58 (75)	32 (76)	1.0
Cachexia domains			
Body mass index (BMI)			
Height,^a^ cm	172 (10)	172 (10)	0.880
Weight,^a^ kg	62 (12)	78 (13)	< 0.001
BMI,^a^ kg/m^2^	20.9 (3.0)	26.4 (3.6)	< 0.001
Weight loss (WL)			
WL,^a^ kg	−6.5 (1.8)	−0.2 (1.6)	< 0.001
WL,^a^ %	−9.7 (3.6)	−0.3 (2.0)	< 0.001
Performance status (PS)			
ECOG‐PS,^c^ *score ≤ 1*	44 (56)	37 (88)	< 0.001
Food intake			
Reduced^a^ (vs. unchanged*)*	59 (76)	11 (26)	< 0.001
Appetite loss			
ESAS Score,^b^ score 0–10	4.0 (3.0, 5.0)	2.0 (0.25, 2.0)	< 0.001
C‐reactive protein (CRP)			
CRP values,^b^ mg/dL	37 (9, 61)	14 (7, 30)	< 0.001
Overall survival (OS)			
Median OS,^d^ months	5 (5, 6)	13 (12, 16)	< 0.001

^a^ Mean (standard deviation) (normal data distribution); Welch two sample *t* test applied to compare groups.

^b^ Median (Quartiles 1 and 3) (outside normal distribution); Wilcoxon rank sum test applied to compare groups.

^c^ Count (percentage); Pearson's Chi‐squared test applied to compare groups.

^d^ Median, 95% confidence interval (CI), log rank sum test applied to compare groups.

### 3.2 Associations of Gut Microbiome Composition, Bacterial Taxa and Microbiome‐Encoded Functions With Cancer Cachexia

**FIGURE 3:**
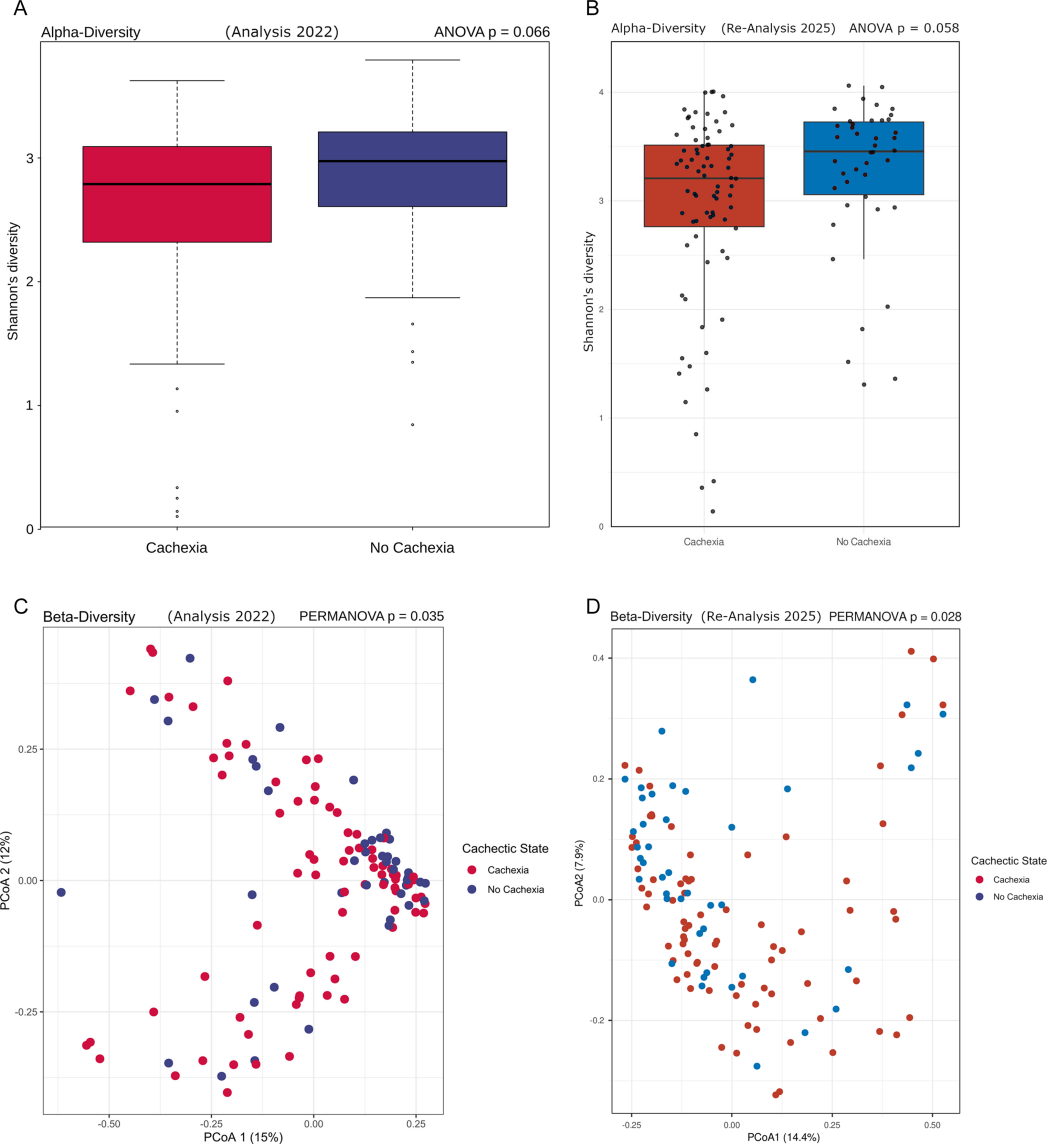
Alpha and beta diversity in the gut metagenome dataset between cachectic (*n* = 78) and noncachectic (*n* = 42) cancer patients based on kraken2 *k*‐mer matching against the NT‐database in the analysis in 2022 and the reanalysis in 2025. Shannon index variation according to cachexia state in pairwise ANOVA analysis in the dataset (A) in 2022 (*p* = 0.066) and (B) in 2025 (*p* = 0.058). Boxes represent IQRs with the median as the horizontal line and the whiskers depicting the lowest and highest values within 1.5‐fold IQR. Principal coordinate analysis of Bray–Curtis distances according to cachexia state in pairwise PERMANOVA analysis with 999 permutations in the dataset (C) in 2022 (*p* = 0.035) and (D) in 2025 (*p* = 0.028). IQR, interquartile range.

**FIGURE 4:**
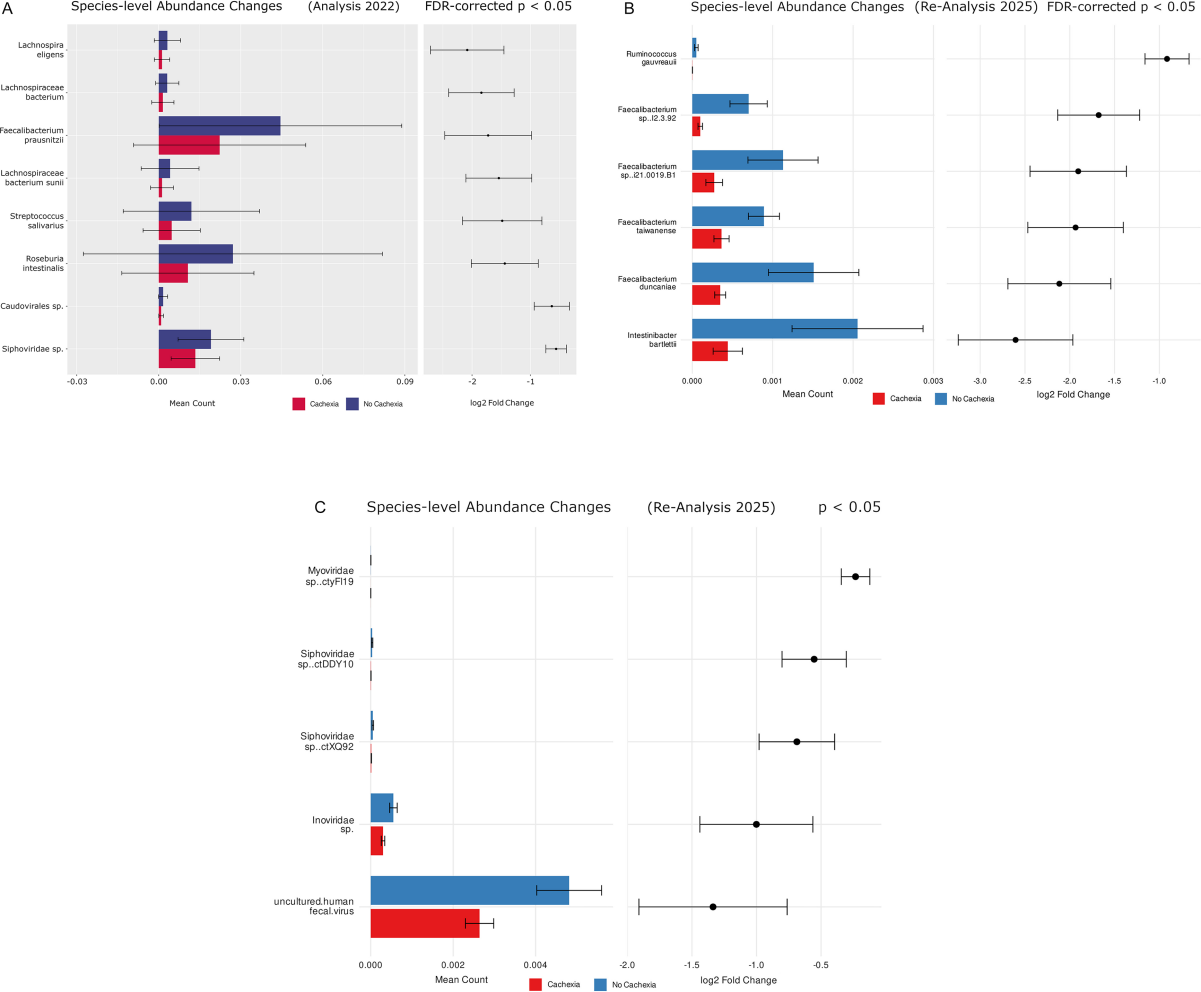
Significant single species‐level abundance changes between cachectic (*n* = 78) and noncachectic (*n* = 42) cancer patients based on kraken2 *k*‐mer matching against the NT database 2022 under the morphology‐based ICTV phage taxonomy (valid until 2022) and the reanalysis 2025 under the revised genome‐based ICTV phage taxonomy. (A) Species with overlapping significance of FDR‐corrected *p* values of < 0.05 in both two‐tailed unpaired *t* test and LinDa analysis for differential abundance in the analysis 2022. (B) Species with overlapping significance of FDR‐corrected *p* values of < 0.05 in both two‐tailed unpaired *t* test and LinDa analysis for differential abundance in the reanalysis 2025. (C) Phage species with nominally significant different abundance (*p* < 0.05 in *t* test) in the reanalysis 2025 under the revised genome‐based ICTV phage taxonomy. Species‐level abundance values represent the relative proportion of read sequence counts related to the total number of read sequence counts after normalization and are presented as mean ± standard deviation. The log2 fold changes display the differences of the log‐transformed relative abundance values. ICTV, International Committee on Taxonomy of Viruses.

Alpha‐diversity was not significantly different between cachectic and noncachectic patients, neither in the analysis of the metagenomics dataset in 2022 (Figure 3A, Shannon index, ANOVA test: *p* = 0.066 [inverse Simpson: *p* = 0.322; Chao1: *p* = 0.060]) nor in the reanalysis in 2025 (Figure 3B, Shannon index, ANOVA test: *p* = 0.058 [inverse Simpson: *p* = 0.128; Chao1: *p* = 0.053]). By contrast, beta‐diversity, indicated by principal coordinates analysis based on Bray‐Curtis distance, was significantly associated with prevalent cachexia in both dataset analyses (Figure 3C, PERMANOVA test: *p* = 0.035, *R* ^2^: 0.16; Figure 3D, PERMANOVA test: *p* = 0.028, *R* ^2^: 0.19). Among taxa derived from read‐based analysis via Kraken2/Bracken, six bacterial species ( *Faecalibacterium prausnitzii* , *Roseburia intestinalis* , *Streptococcus* species and three *Lachnospiraceae* species) in the analysis in 2022 (Figure 4A and Table S1A) and six bacterial species (four *Faecalibacterium species*, *Ruminococcus gauvreauii* , *Intestinibacter bartlettii*) in the re‐analysis in 2025 (Figure 4B and Table S1B) exhibited significantly lower abundance in cachectic patients with FDR‐corrected *p* < 0.05 in both *t*‐test and LinDA analysis. In the first analysis in 2022, there were a further 21 species (Table S2A) and in the reanalysis in 2025, there were 45 species (Table S2B) that showed nominally significant different abundance between cachectic and noncachectic cancer patients. In studying associations of microbiome functions, we found a total of 43 cachexia‐associated enriched or depleted microbiome‐encoded KO groups (Tables S3A–S3C). Remarkably, we observed positive associations with enriched gut microbial β‐glucuronidases (KO1195) (*p* < 0.05 in LinDA analysis, Table S3A) and depleted major facilitator family efflux pumps (O6902) (*p* < 0.05 in *t*‐test and LinDA analysis; Table S3C), suggesting that cancer cachexia associated with a reduced functional gut microbiome capability for the elimination of toxic compounds.

### 3.3 Alterations of Gut Bacteriophages in Read‐Based Analysis Associated With Cancer Cachexia

Among taxa derived from read‐based analysis with Kraken2/Bracken *k*‐mer matching against the NT‐database in 2022 under the morphology‐based ICTV phage taxonomy, we found that two double‐stranded (ds) DNA bacteriophage species (*Siphoviridae* and *Caudovirales* species) exhibited lower abundance with overlapping significance (FDR‐corrected *p* < 0.05 in both *t*‐test and LinDA analysis) in cachectic patients (Figure 4A and Table S1A). In addition, further ds‐DNA bacteriophages, namely, *Myoviridae* species, and single‐stranded (ss) DNA bacteriophages comprising the *Inoviridae* and *Microviridae* families, associated nominally significant with a reduced abundance in the cachexia cohort (*p* < 0.05 in *t* test, Table S2A). In the reanalysis in 2025, which applied the revised genome‐based ITCV taxonomy for phage annotation and characterization, we could not identify bacteriophage species from read‐based analysis by Kraken2/Bracken that showed an overlapping, significantly different abundance with FDR‐corrected *p* < 0.05 in both *t*‐test and LinDA analysis between cachectic and noncachectic patients. However, several bacteriophages showed nominally significant reduced abundance (*p* < 0.05 in t‐test) in cachectic patients (Figure 4C). Among these, there were three ds‐DNA bacteriophages (*Myoviridae species* CtyF119, *Siphoridae species* CtDDY10 and *Siphoridae species* CtXQ92) and one ss‐DNA bacteriophage (*Inoviridae species*) (Table S2B).

### 3.4 Alterations of Gut Bacteriophages in Assembly‐Based Analysis Associated With Cancer Cachexia

MetaviralSPAdes [22] was used to de novo assemble viral contigs and to identify viral genomes from metagenomics dataset generated in 2022 under the morphology‐based ITCV taxonomy and in 2025 under the revised genome‐based ICTV taxonomy. In 2022, we obtained 4.184 de novo assembled contigs, of whom 3.698 contigs (88.4%) could be assigned to a species (Table S4). Among the most abundant contigs, a multitude of bacteriophage species could be identified and assigned to the species *Bacteriophages*, *Caudovirales*, *CrAss‐like viruses*, *Inoviridae*, *Microviridae*, *Myoviridae*, *Podoviridae* and *Siphoviridae* (Table S4). Most significantly differential covered contigs (180 of 204 in *t* test; 89 of 89 in LinDA analysis) could be assigned to species by BLASTn mapping against the NT‐database and showed significantly lower abundance of *Caudovirales*, *Myoviridae* and *Siphoviridae* (*p* < 0.05 in *t*‐test and LinDa analysis) and *Bacteriophages and Microviridae* (*p* < 0.05 in *t* test) in the cachexia cohort (Tables S5 and S6). By applying VIBRANT to our first analysis in 2022 to incorporate annotation information into lifestyle categorization, the majority of identified phage species (12 of 13 in *t* test; 4 of 6 in LinDA analysis) were classified as lytic phage genomes, specifically belonging to *Caudovirales*, *Siphoviridae*, *Bacteriophage* sp., *Myoviridae* and *Microviridae* phage species (Table S7). In our re‐analysis in 2025, which aimed to test for the reproducibility of our results under the 2022 revised genome‐based ITCV taxonomy on our metagenomics dataset inferred from the NT‐database in 2025, we obtained 4.209 de novo assembled contigs, of whom 3.916 contigs (93.0%) could be assigned to a total of 704 species, from which 39 were bacteriophage or viral species (Table S8). Most significantly differential abundant contigs (195 of 208 in *t* test, 280 of 315 in LinDa analysis) could be assigned to a species by BLASTn mapping against the NT‐database. Among these, there were two ds‐DNA bacteriophages (*Myoviridae species* and *Siphoridae species*) and one ss‐DNA bacteriophage (*Inoviridae species*) that showed nominally significant reduced abundance (*p <* 0.05 in *t* test and LinDa analysis) in the cachexia cohort (Tables S9 and S10).

### 3.5 Machine Learning Models for Prediction of Cachectic State

**FIGURE 5:**
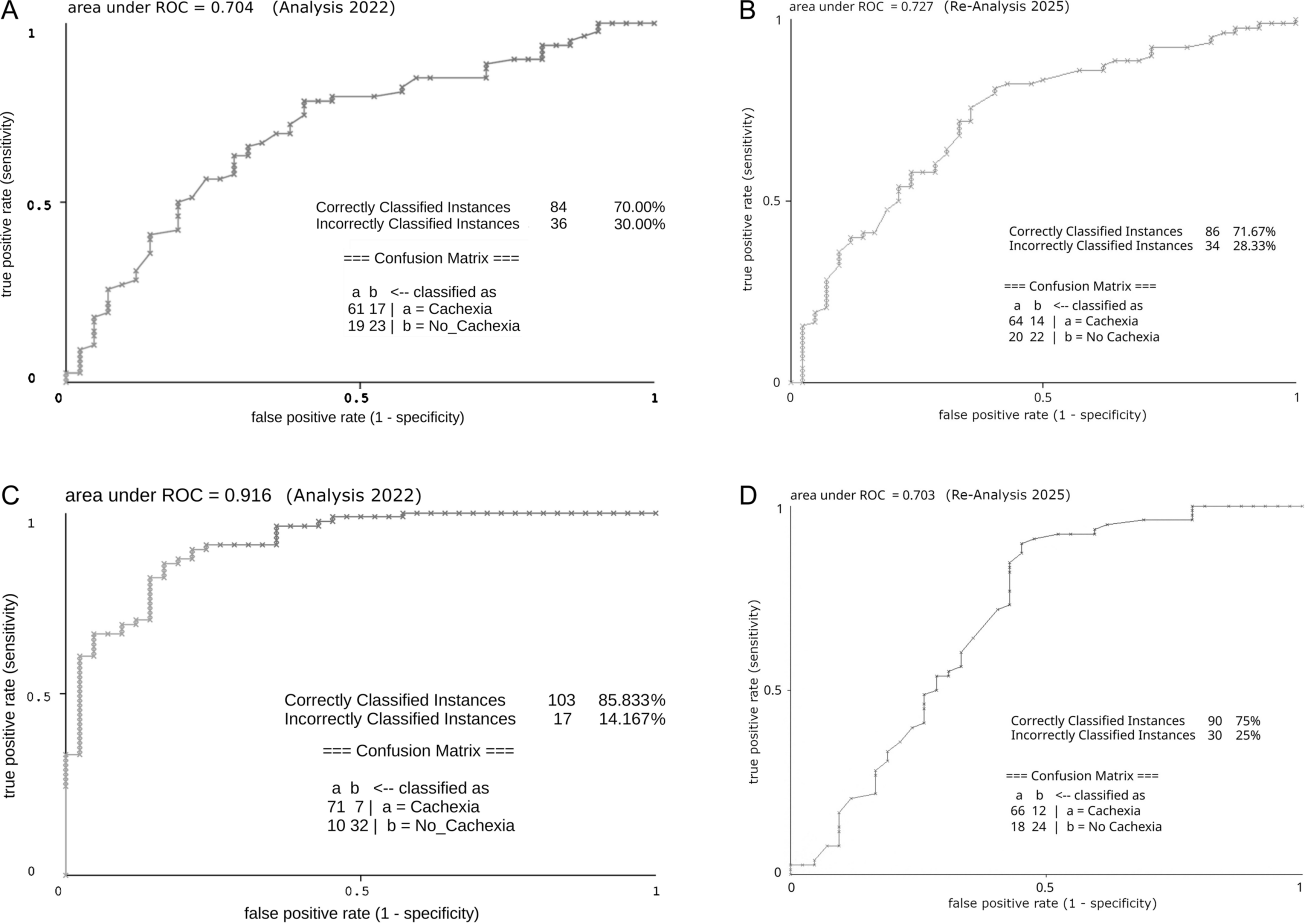
ROC curves of random forest models for classifying cancer patients (*n* = 120) between cachectic (*n* = 78) and noncachectic (*n* = 42) state. (A) ROC based on the metagenomics dataset inferred 2022 by the NT database. (B) ROC based on the metagenomics dataset inferred 2025 by the NT database. (C) ROC inferred by *de novo* assembled viral contigs generated in 2022 under the morphology‐based ICTV phage taxonomy (valid until 2022). (D) ROC inferred by de novo assembled viral contigs generated in 2025 under the revised genome‐based ICTV phage taxonomy. Lists below ROC curves show the confusion matrix and accuracy values of the model. ICTV, International Committee on Taxonomy of Viruses; ROC, receiver operating characteristic.

Random forest (RF) models were trained to discriminate between cachectic and noncachectic patients using taxa derived from Kraken2 *k*‐mer mapping against the NT‐database in 2022 under the morphological‐based ITCV taxonomy and in 2025 under the revised genome‐based ITCV taxonomy. Prior to RF model training, a feature selection was performed, and only species that showed nominally significant differential abundance in t‐test and/or LinDA analysis (*p* < 0.05) were used as features for training. Based on the 2022 and 2025 metagenome datasets, the RF models predicted cachexia, respectively, with 70.0% and 71.7% correctly classified instances and a ROC‐AUC of 0.704 and 0.727 (Figure 5A,B). Notably, in the prediction‐oriented RF models using the metagenomics dataset from 2022 and 2025 for classification between cachectic and noncachectic patients; ds‐DNA bacteriophages; such as *Caudovirales* and *Siphoviridae* species according to the morphological‐based ICTV taxonomy (Table S11A); and ds‐DNA bacteriophages such as *Myoviridae species* CtyF119 and *Siphoridae species* CtDDY10 according to the revised genome‐based ICTV taxonomy, were among the top predictors (Table S11B). Training a RF model on the abundance species derived from de novo assembled viral contigs in 2022 and 2025 yielded increased predictive performance with, respectively, 85.8% and 75% correctly classified instances and a ROC‐AUC of 0.916 and 0.703 (Figure 5C,D). The RF models were evaluated using tenfold cross‐validation, and detailed performance metrics are provided in Tables S12A and S12B.

## 4 Discussion

Our work revealed important perturbations in the gut microbiome of cachectic patients at diagnosis of advanced‐stage, metastatic gastrointestinal cancers. The most important finding was a previously unknown link between the gut phageome and cancer cachexia. Gut bacteriophages proved to be key features for distinguishing cachectic from noncachectic patients, consistently across read‐based, assembly‐based, significance‐based statistical and prediction‐oriented ML approaches. Moreover, the phage signal was concordantly reproducible with reference to the morphology‐based (valid until 2022) and the recently revised genome‐based ICTV phage taxonomy in 2022‐ and 2025‐generated metagenomics datasets. However, the findings presented here are observational; as such, they are exploratory and intended to stimulate future work to explore the underlying biology, biomedical relevance and therapeutic potential of the microbiome changes identified in this study.

Previous clinical and animal studies have linked changes of the bacterial composition and/or microbial functions in gut to cancer cachexia [6, 7, 8, 9, 10, 26]. Although translating animal study findings to complex human diseases is limited, animal models are important to improve the functional understanding of findings observed in human studies. Consistent with reported gut microbiome profiles in human cancer cachexia [7, 8, 9], we found that beta‐diversity (microbial community structure), but not alpha‐diversity, and *Faecalibacterium species* abundance, significantly differed between cachectic and noncachectic patients. In contrast, we could not replicate several reported shifts of bacterial species (e.g., *Prevotella copri* , *Lactobacillus*, *Megamonas*, *Peptococcus*, *Veillonella*, *Enterobacteriaceae* and *Blautia*) [6, 7, 8, 9]. Moreover, we found previously unreported shifts of bacterial species, with some overlaps and differences, based on the metagenomics datasets generated in 2022 ( *F. prausnitzii* , *R. intestinalis* , *Streptococcus species* and *Lachnospiraceae species*) and 2025 (*Faecalibacterium species*, *R. gauvreauii* and *Intestinibacter bartlettii*). Altogether, clinical cohort studies, including ours, indicate gut microbiome changes in human cancer cachexia, but specific microbial findings remain inconsistent. The cross‐cohort variability of research findings may come from sources such as small sample size, differences in study design, patient selection and/or methodologies applied; however, we found some intracohort variability of bacterial findings, based on the same sequencing data but metagenomic datasets generated in 2022 and 2025. This suggests that some inconsistencies in replicating the same result could be due to increasing inputs of sequencing data and/or dynamic changes of reference and/or assembled genomes in databases for characterizing microbial species [17, 18] and that updates to existing analyses may narrow down robust microbiome findings [S23].

Previous studies have reported several alterations of gut microbial functions in human cancer cachexia, mostly affecting metabolic pathways of carbohydrates, sugars, nucleotides, amino acids and short‐chain acids [6, 7, 8, 9, 10]. Our results point out a reduced gut microbial capacity to eliminate toxic compounds by enriched microbial β‐glucuronidase [S24] and depleted bacterial efflux pumps [S25, S26]. Because both mechanisms are widespread within bacterial populations, this finding may extend beyond cancer cachexia itself and supports research on modulating the efficacy and toxicity of anticancer therapy by distinct gut microbiomes [27, 28].

Our most noticeable finding is the previously unknown close link between decreased levels of DNA bacteriophage species in gut and human cancer cachexia. The significance of the phage signal was stronger under the morphology‐based ICTV taxonomy (valid until 2022) but reproducible in the reanalysis 2025 under the genome‐based ICTV revision [15, 16]. Both analyses outlined that decreased abundances affected predominantly dsDNA phages (2022: *Caudovirales*, *Siphoviridae*; 2025: *Myoviridae*, *Siphoridae*) but also very low‐abundant ssDNA phage species (2022: *Inoviridae*, *Microviridae*; 2025: *Inoviridae*). Moreover, purely data‐driven ML models ranked dsDNA phages as top predictors for cachexia state (2022: *Caudovirales*, *Siphoviridae*; 2025: *Myoviridae*, *Siphoridae*). Moreover, ML models yielded highest accuracy when only phage contigs were taken into account, reaching up to 85.8% correctly classified instances and area under the ROC curve values up to 0.916. Notably, we found no link to cachexia for widespread *crAss‐like* dsDNA phages, now forming the order *Crassviridae*, comprising > 85% of all gut phages [S27, S28]. Established *crAss‐like* phage populations in gut are highly stable [S29, S30], likely because they predominately replicate as phage‐plasmid [S31]; disease‐associated changes in the *crAss‐like* gut phage population appear to be overall rare [29] but have been observed in autoimmune [S32], metabolic [S33] and inflammatory bowel disease [S34]. The most relevant change in the ICTV classification, however, is the abolishment of the large morphological family *Siphoviridae* and order *Caudovirales* [15, 16], which both essentially contributed to the significance of our phage signal. The ICTV revision focuses on reconstructing evolutionary relationships, but the reorganization led to a significant floating of phage subfamilies and genera, leaving behind various orphaned phage subfamilies, formerly assigned to *Siphoviridae* or *Caudovirales*, without a designated family or order in the new class *Caudoviricetes* [15, 16, 17, 18, 19]. Although our main findings and conclusions remain unchanged from the changed ICTV framework, our study suggests a possible impact of the revised ICTV standard on results of studying the gut phageome in human disease. It is also of note that the abolishment of *Siphoviridae* and *Caudovirales* limits the exchange and cross‐trial comparability of research data between previous and future studies.

Perturbations of the gut microbiome, termed dysbiosis, affect various human diseases, but typically, the gut phageome is increased, rather than decreased, as seen in this study [12]. Furthermore, reported disease‐associated increases often implicate an expansion of *Caudovirales* taxa and *Siphoviridae* families [12], for example, in obesity, diabetes and inflammatory bowel diseases [S7–S9, S34]. Mechanistically, increased gut phage abundance predominantly results from prophage induction and/or higher lytic phage activity, whereas decreased phage abundance could simply reflect reduced bacterial host abundance [12], [S35–S36]. Because the majority of human gut phages are prophages and exhibit a temperate behaviour, their decline is likely to coevolve with the decline of the total prophage‐carrying bacterial load [30], [S37]. However, in our study, the reduced phage species predominantly exhibited a lytic lifestyle, possibly because specific bacterial subpopulations (e.g., Faecalibacterium) are reduced, and the lytic phages that infect these bacteria also decline due to their limited host range [13], [S38]. In fact, we observed a co‐occurring phage‐bacteria decline, but especially the decline of lytic gut phages, could also result from increased wash‐out, for example, due to reduced phage adherence in the mucus layer [31]. Further, reduced phage counts in the gut lumen, especially for those exhibiting a lytic lifestyle, could also result from increased phage translocation into the submucosa. Beyond the gut lumen, ample amounts of gut phages are present in the gut mucosa and interact with the innate and adaptive immunity of the human host [32], and ~3 × 10^7^ gut phages translocate daily across human gut epithel cells and into the human circulation [33]. However, our study is limited to stool samples, and from our cross‐sectional shotgun metagenomics data, we cannot discern DNA packaged in phage particles from prophages in bacterial genomes and/or estimate phage‐bacteria pairs. Whether the reduced gut phage signals in cancer cachexia stem from prophages, free phage particles or both, additional investigations on how interconnected intraintestinal and extraintestinal phageomes are, and quantitative measurements of the total bacterial load (e.g., by using qPCR that targets the universal bacterial 16S rRNA gene), remain queries for future work.

In animal models, faecal virome transplantation (FVT) showed potential for treatment of obesity, diabetes and immune responses [S39–41]. Apart from FVT, which targets broadly the gut bacteriome, more specific phage therapy has shown clinical benefit in treating pathogenic and/or drug‐resistant bacterial infections [34]. Our findings could lead to the conclusion to alleviate cancer cachexia by targeted phage therapy of declined gut phage species. However, the expansion of *Caudovirales* bacteriophages in gut lumen and mucosa associates with local inflammation and colitis in animal models and humans [S42–43], and transplantation of bacteriophages from ulcerative colitis patients exacerbates colitis in animal models [S44]. These findings demand the consideration of clinical implications of therapeutic phage applications [35], especially in the context of reported systemic phage‐directed immune responses to phage‐based therapeutics in humans, which could be beneficial or detrimental [27, 36]. Although our findings suggest that the restoration of normal gut phage levels could be conceptually interesting, future research should focus on mechanisms linking gut phages and bacteria to a cachectic phenotype in cancer patients [12, 14, 37], and whether identified mechanisms provide target candidates that could translate into proof of concept, efficacy and safety assessments in clinical trials [38], [S45].

Our study has limitations. First, the sample size was limited, and the data from this exploratory, single‐centre study will need confirmatory testing in a larger, multicentre cohort. Second, our case–control design used cancer patients without cachexia as control group, which isolates results to cachexia as opposed to other phenomena associated with cancer. Future research may include household partners and healthy controls to better assess environmental effects and/or early effects in the cachexia trajectory. Third, our cross‐sectional design does not capture longitudinal changes. However, because intraindividual and interindividual variation of the gut phageome can confound phage signal detection [25], [S29, S36], longitudinal data in much larger cohorts will be required to explore confounding effects from the variation of phage populations. Fourth, covariables, such as diet, medication and cancer type, may affect the gut microbiome composition [12], [S36, S46]. However, covariables, except for cachexia domains, showed nearly similar within‐group distribution without significant between‐group differences between the cachectic and noncachectic patient groups. Further, the hierarchical clustering analysis of samples revealed no association between the abundance of certain microbial species and the cancer type, for neither the entire cohort, cachexia subgroup, nor noncachexia subgroup, nor in the analysis 2022 or reanalysis 2025. The overall small sample size, especially the small subgroup sizes for cancer types, together with high numbers of microbiome features per sample and the unknown effect sizes of intraindividual and interindividual variation in our metagenomic datasets, limited us to produce further meaningful data with respect to potential confounders, especially to rule out a confounding effect of the cancer type. To study the effect of the cancer type in human cancer cachexia in more detail, much larger, multicentric and longitudinally designed studies in homogeneous cancer patient groups and/or with substantially increased numbers for different cancer types will be required. Fifth, we defined cancer cachexia by BMI‐adjusted WL. This widely used criterion agreed upon international consensus [1] correlated with several other cachexia domains (CRP level, appetite loss, food intake, ECOG‐PS and OS). Although this in part validates our definition of cachexia, our analysis would have benefitted from additional computed tomography‐based muscle mass measurements and/or skeletal muscle function tests to measure sarcopenia [S47]. Finally, characterization of the gut phageome will be improved as more phage genomes are identified and bioinformatics tools improve. Further, virus‐like‐particle separation, upfront viral library preparation, long‐read techniques, multicompartment metagenomics, transcriptomics and reverse transcription steps might reveal further associations for DNA and RNA phages, potentially including functional data on expression and intrabody distribution of gut phage genes [39], [S48]. Also, additional quantitative data based on qPCR techniques will be required to confirm changes in relative phage abundance identified by means of metagenomics approaches. Collectively, these future investigations could provide testable hypotheses and/or targets to investigate causality questions and/or the clinical significance of modulating the gut phageome to management of cancer cachexia.

Strengths of the study include a high sensitivity of shotgun sequencing for phage‐inclusive profiling at ~20 million reads per sample, the use of state‐of‐the‐art tools for bacteriophage identification and de novo assembly of viral contigs, and a strict control of false discovery and overfitting in statistical (e.g., overlapping significance of different approaches for taxon‐based selection) and ML methods (e.g., tenfold cross‐validation, meta‐classifier approach and cost‐sensitive base classifiers for community‐level separation of cachexia state) [20, 21, 22, 23, 24, 25]. Identifying bacteriophages as both highly significant and highly predictive species by complementary methods that differentially process the data supports a real phage signal, rather than a stochastic or spurious result [25, 40]. Furthermore, the phage signal was replicable under both the morphology‐based and revised genome‐based ICTV phage taxonomy [15, 16, 17, 18, 19]. However, we report early hypothesis‐generating data. Because there are no comparable studies with prospective data on gut viruses in this still emerging field, confirmatory testing is warranted in an independent prospective multicentre validation. Nonetheless, given the limited human data on the gut microbiome in cancer cachexia research, our dataset may provide a resource for the exchange of research data between previous and future studies in an important field of cancer patient care.

## 5 Conclusions and Future Directions

This study is the first to show, beyond alterations in the gut bacterial composition and microbiome functionality, an association between decreased intestinal DNA bacteriophage species and human cancer cachexia. This finding expands the scope for basic, translational and clinical microbiome‐targeted research in an area of significant unmet medical need. Association does not imply causality, and we hope that our findings pave the way for future mechanistic work to explore the underlying biology, biomedical relevance and therapeutic potential of the microbiome alterations identified in this study. Specifically, future work should explore whether the decreased gut phage signals stem from prophages, free phage particles or both, whether the decreased phage abundance reflects decreased bacterial host abundance, and/or increased phage wash‐out or translocation into the body, whether associations include further DNA and/or RNA phages, whether phage abundance changes occur causally, in response to cachexia, or both, and whether gut bacteriome and/or phageome modulation has potential to counteract cancer cachexia.

## Funding

The work was supported by a grant (#3465) awarded to Axel Stang by Asklepios Proresearch, Asklepios Hospitals Hamburg, Germany.

## Ethics Statement

This prospective cohort study was approved by Ärztekammer Hamburg (Protocol Number: V5649, Date: 23 October 2017) and is registered in the German Cancer Society Study Box (Registration Number: ST‐U069; https://www.studybox.de). All patients provided written informed consent prior to study inclusion for scientific evaluation of pseudonymized data. The studies were conducted in accordance with the ethical standards laid down in the 1964 Declaration of Helsinki and its later amendments.

## Conflicts of Interest

The authors declare no conflicts of interest.

## Supporting information


**Figure S1:** (A–F) Hierarchical clustering of the abundance of certain gut microbial species with cancer type in the entire cohort (*n* = 120), cachectic (*n* = 78) and non‐cachectic (*n* = 42) cancer patients in the metagenomic datasets inferred by the NT database in 2022 and 2025. Heatmaps show no association to the abundance of certain in the analysis in 2022 and 2025 for the entire cohort (A, B) and for separate analyses of cachectic patients (C, B) or non‐cachectic patients (E, F). Heatmaps were generated by applying a distance threshold of 0.6 in hierarchical clustering using the linkage method with Euclidean distance as distance metrics. Colour scale represents the normalized mean count of relative abundance for species with ≥ 0.1% relative mean abundance in the whole dataset.

**Figure S1B:** Supplementary Information.

**Figure S1C:** Supplementary Information.

**Figure S1D:** Supplementary Information.

**Figure S1E:** Supplementary Information.

**Figure S1F:** Supplementary Information.

**Table S1A:** Species derived from 1.312 taxa inferred by read‐based mapping with Kraken/Bracken2 *k*‐mer matching against the NT‐database in 2022 under the morphology‐based ICTV phage taxonomy that showed overlapping significance for differential abundance by FDR‐corrected *p* values of < 0.05 in both two‐tailed unpaired t‐test (pairwise comparison of non‐transformed mean abundance values) and LinDA analysis (linear regression of log2‐transformed data) between cachectic (*n* = 78) compared to non‐cachectic cancer patients (*n* = 42). Species abundance is represented as compositional data, expressed as relative mean abundance (dimensionless proportion of the total metagenomics dataset) for each taxon, and transformed using the centered log‐ratio transformation before linear regression was applied.

**Table S1B:** Species derived from 1.513 taxa inferred by read‐based mapping with Kraken/Bracken2 *k*‐mer matching against the NT‐database in 2025 under the revised genome‐based ICTV phage taxonomy that showed overlapping significance for differential abundance by FDR‐corrected *p* values of < 0.05 in both two‐tailed unpaired t‐test (pairwise comparison of non‐transformed mean abundance values) and LinDA analysis (linear regression of log2‐transformed data) between cachectic (*n* = 78) compared to non‐cachectic cancer patients (*n* = 42). Species abundance is represented as compositional data, expressed as relative mean abundance (dimensionless proportion of the total metagenomics dataset) for each taxon, and transformed using the centered log‐ratio transformation before linear regression was applied.

**Table S2A:** Species derived from 1.312 taxa inferred by read‐based mapping with Kraken/Bracken2 *k*‐mer matching against the NT‐database in 2022 under the morphology‐based ICTV phage taxonomy that showed nominal significance for differential abundance (*p* < 0.05) in two‐tailed unpaired t‐test (pairwise comparison of non‐transformed mean abundance values) between cachectic (*n* = 78) compared to non‐cachectic cancer patients (*n* = 42). Species abundance is expressed as relative mean abundance (dimensionless proportion of the total metagenomics dataset) for each taxon.

**Table S2B:** Species derived from 1.513 taxa inferred by read‐based mapping with Kraken/Bracken2 *k*‐mer matching against the NT‐database in 2022 under the morphology‐based ICTV phage taxonomy that showed nominal significance for differential abundance (*p* < 0.05) in two‐tailed unpaired t‐test (pairwise comparison of non‐transformed mean abundance values) between cachectic (*n* = 78) compared to non‐cachectic cancer patients (*n* = 42). Species abundance is expressed as relative mean abundance (dimensionless proportion of the total metagenomics dataset) for each taxon.

**Table S3A:** Functional KEGG annotations from microbial‐encoded genes of stool samples that were significantly different enriched (*p* < 0.05) in cachectic (*n* = 78) compared to non‐cachectic cancer patients (*n* = 42) in LinDA analysis.

**Table S3B:** Functional KEGG annotations from microbial‐encoded genes of stool samples that were significantly different enriched (*p* < 0.05) in cachectic (*n* = 78) compared to non‐cachectic cancer patients (*n* = 42) in t‐test.

**Table S3C:** Functional KEGG annotations from microbial‐encoded genes of stool samples that were significantly different enriched (*p* < 0.05) in cachectic (*n* = 78) compared to non‐cachectic cancer patients (*n* = 42) in both t‐test and LinDA analysis.

**Table S4:** De novo annotated species contigs by BLASTn mapping against the NT‐database in 2022 under the morphology‐based ICTV phage taxonomy for the entire patient cohort (*n* = 120).

**Table S5:** jcsm70324‐sup‐0015‐Supplementary_TableS5.xlsx. *De novo* annotated species contigs by BLASTn mapping against the NT‐database in 2022 under the morphology‐based ICTV phage taxonomy that were significantly different in abundance (*p* < 0.05) in cachectic (*n* = 78) compared to non‐cachectic cancer patients (*n* = 42) in t‐test.

**Table S6:** jcsm70324‐sup‐0016‐Supplementary_TableS6.xlsx. *De novo* annotated contigs by BLASTn mapping against the NT‐database in 2022 under the morphology‐based ICTV phage taxonomy that were significantly different in abundance (*p* < 0.05) in cachectic (*n* = 78) compared to non‐cachectic cancer patients (*n* = 42) in LinDA analysis.

**Table S7:** VIBRANT life style qualification of *de novo* assembled species contigs annotated by BLASTn mapping against the NT‐database in 2022 under the morphology‐based ICTV phage taxonomy that were significantly lower (*p* < 0.05) in cachectic (*n* = 78) compared to non‐cachectic cancer patients (*n* = 42) in t‐test and LinDA analysis.

**Table S8:** jcsm70324‐sup‐0018‐Supplementary_TableS8.xlsx. *De novo* annotated species contigs by BLASTn mapping against the NT‐database in 2025 under the revised genome‐based ICTV phage taxonomy for the entire patient cohort (*n* = 120).

**Table S9:** jcsm70324‐sup‐0019‐Supplementary_TableS9.xlsx. *De novo* annotated species contigs by BLASTn mapping against the NT‐database in 2022 under the morphology‐based ICTV phage taxonomy that were significantly different in abundance (*p* < 0.05) in cachectic (*n* = 78) compared to non‐cachectic cancer patients (*n* = 42) in t‐test.

**Table S10:** jcsm70324‐sup‐0020‐Supplementary_TableS10.xlsx. *De novo* annotated species contigs by BLASTn mapping against the NT‐database in 2025 under the revised genome‐based ICTV phage taxonomy that were significantly different in abundance (*p* < 0.05) in cachectic (*n* = 78) compared to non‐cachectic cancer patients (*n* = 42) in LinDA analysis

**Table S11A:** Classifier importance (≥ 0.30) in random forest‐based machine learning models running under metagenomics data of taxa inferred by read‐based mapping with *k‐mer* matching from the NT‐database in 2022 under the morphology‐based phage taxonomy as input features for classification between cachectic (*n* = 78) and non‐cachectic cancer patients (*n* = 42). Classifier importance was assessed using the metrics mean decrease in impurity and the number of nodes in which the predictor appeared (Gini importance). Cancer patients (n = 42).

**Table S11B:** Classifier importance (≥ 0.30) in random forest‐based machine learning models running under metagenomics data of taxa inferred by read‐based mapping with *k‐mer* matching from the NT‐database in 2025 under the revised genome‐based ICTV phage taxonomy as input features for classification between cachectic (*n* = 78) and non‐cachectic cancer patients (*n* = 42). Classifier importance was assessed using the metrics mean decrease in impurity and the number of nodes in which the predictor appeared (Gini importance).

**Table S12A:** Performance and classification metrics of random forest‐based machine learning models running under the metagenomics data inferred from the NT‐database in 2023 and the morphology‐based ICTV phage taxonomy for classification between cachectic (*n* = 78) and non‐cachectic cancer patients (*n* = 42). Performance parameters shown are based on 10‐fold cross‐validation and a meta‐classifier approach to make base classifier cost‐sensitive.

**Table S12B:** Performance and classification metrics of random forest‐based machine learning models running under the metagenomics data inferred from the NT‐database in 2025 and the revised genome‐based ICTV phage taxonomy for classification between cachectic (*n* = 78) and non‐cachectic cancer patients (*n* = 42). Performance parameters shown are based on 10‐fold cross‐validation and a meta‐classifier approach to make base classifier cost‐sensitive.

**Data S1:** Supplementary Information.

## Data Availability

All data needed to evaluate the conclusions in the paper are provided within the manuscript and its Supporting Information files. Raw data are available on the EGA archive (https://ega‐archive.org/, under accession number EGAC00001003186; DAC portal: MiBiTuKa). The codes, software packages and databases used for the analyses reported in this manuscript are publicly available. The NBCI nucleotide selection databases used for differential abundance analysis can be found online (at https://ftp.ncbi.nlm.nih.gov/blast/db/FASTA/nt.gz). The sequences and annotations for functional analyses that were mapped with BWA‐MEM against the ICG human reference catalogue are available from the China National GeneBank repository (at https://db.cngb.org/microbiome/genecatalog/genecatalog_human/). Software packages include Weka (at https://waikato.github.io/weka‐wiki/downloading), kraken2 (at https://github.com/DerrickWood/kraken2), bracken (at https://github.com/jenniferlu717/Bracken), SPAdes at (https://github.com/ablab/spades), Vibrant (at https://github.com/AnantharamanLab/VIBRANT), LinDA (at https://github.com/zhouhj1994/LinDA or https://cran.r‐project.org/web/packages/MicrobiomeStat/index.html), Blastn (at https://blast.ncbi.nlm.nih.gov/doc/blast‐help/downloadblastdata.html) and BWA‐MEM (at https://sourceforge.net/projects/bio‐bwa/files/). All custom scripts used to generate results and plots are available from the github repository 554 (https://github.com/Connexome/NCOMMS‐22‐23700/; at URL https://www.nature.com/articles/s41467‐022‐32718‐x). Further information and requests for data, codes, software packages and resources should be directed to and will be fulfilled by A. Stang and M. Seifert.
