# Supplementary figures and images for: Lowered Abundance of Gut Bacteriophage Species Is Associated With Human Cancer Cachexia
Source: J Cachexia Sarcopenia Muscle. 2026 Jun 7;17(3):e70324. doi: 10.1002/jcsm.70324 (PMC13243887; doi:10.1002/jcsm.70324)

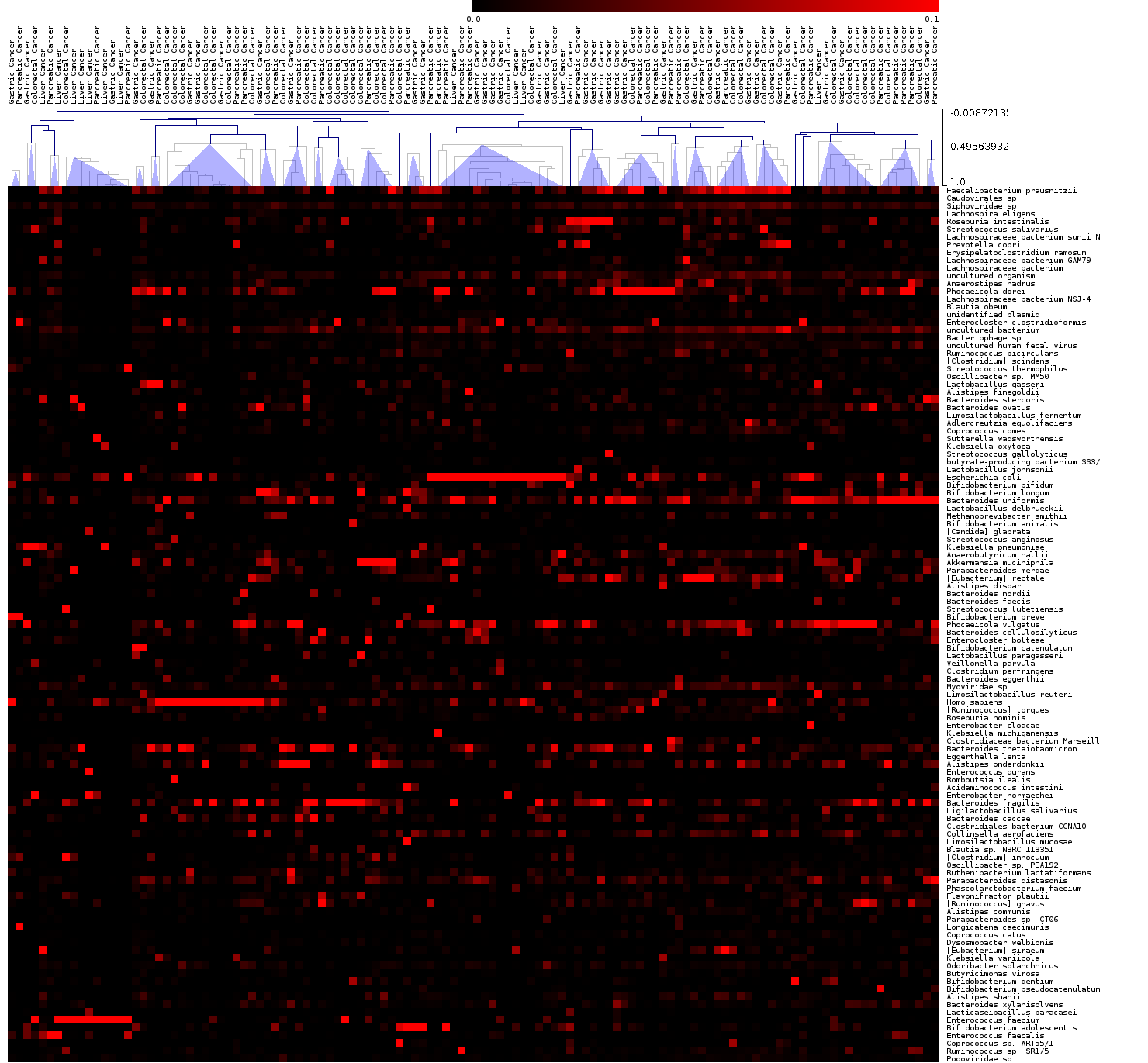

Supplement: Supplementary file 1 — Figure S1: (A–F) Hierarchical clustering of the abundance of certain gut microbial species with cancer type in the entire cohort (n = 120), cachectic (n = 78) and non‐cachectic (n = 42) cancer patients in the metagenomic datasets inferred by the NT database in 2022 and 2025. Heatmaps show no association to the abundance of certain in the analysis in 2022 and 2025 for the entire cohort (A, B) and for separate analyses of cachectic patients (C, B) or non‐cachectic patients (E, F). Heatmaps were generated by applying a distance threshold of 0.6 in hierarchical clustering using the linkage method with Euclidean distance as distance metrics. Colour scale represents the normalized mean count of relative abundance for species with ≥ 0.1% relative mean abundance in the whole dataset. [file JCSM-17-e70324-s023.tiff]

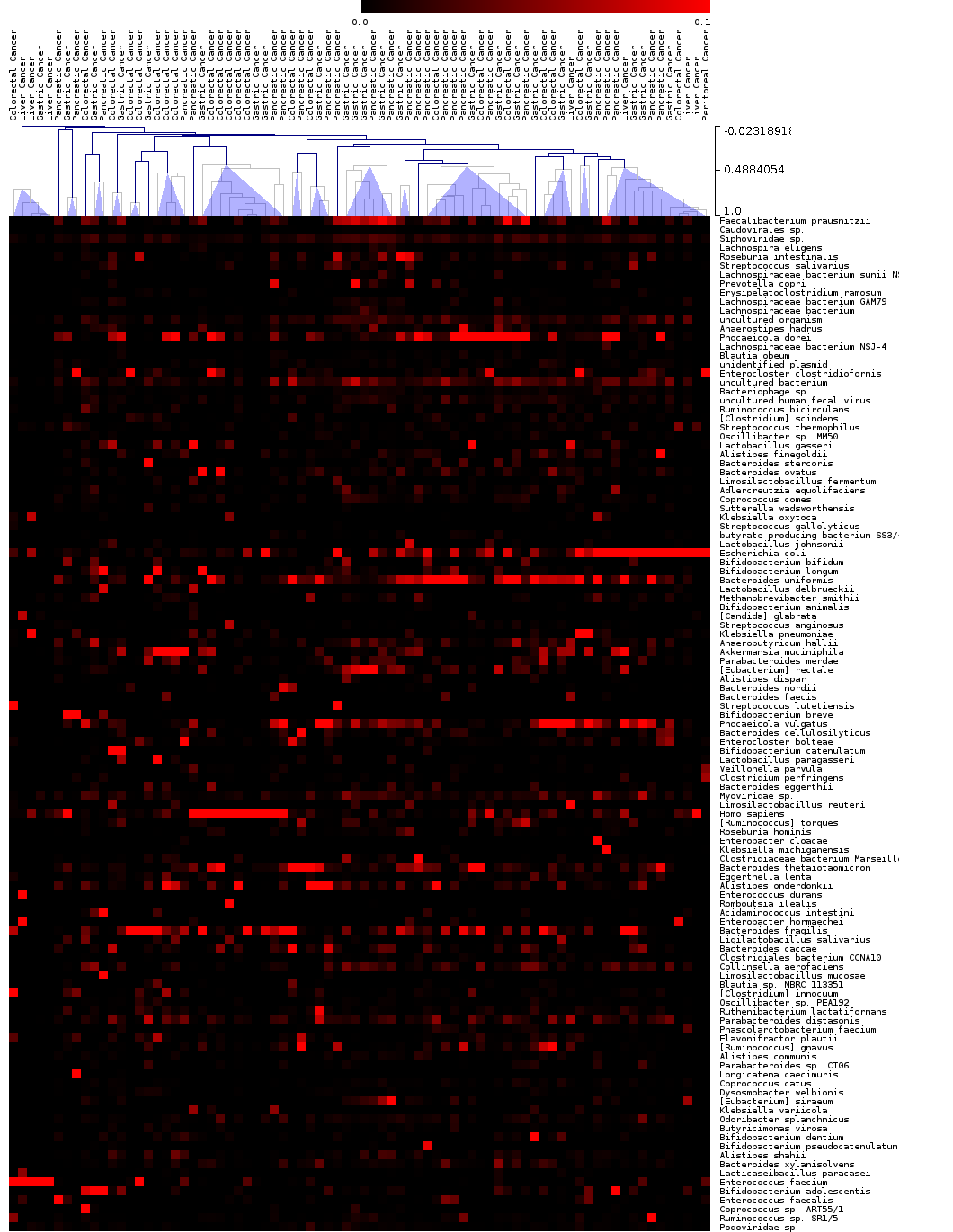

Supplement: Supplementary file 2 — Figure S1B: Supplementary Information. [file JCSM-17-e70324-s002.tiff]

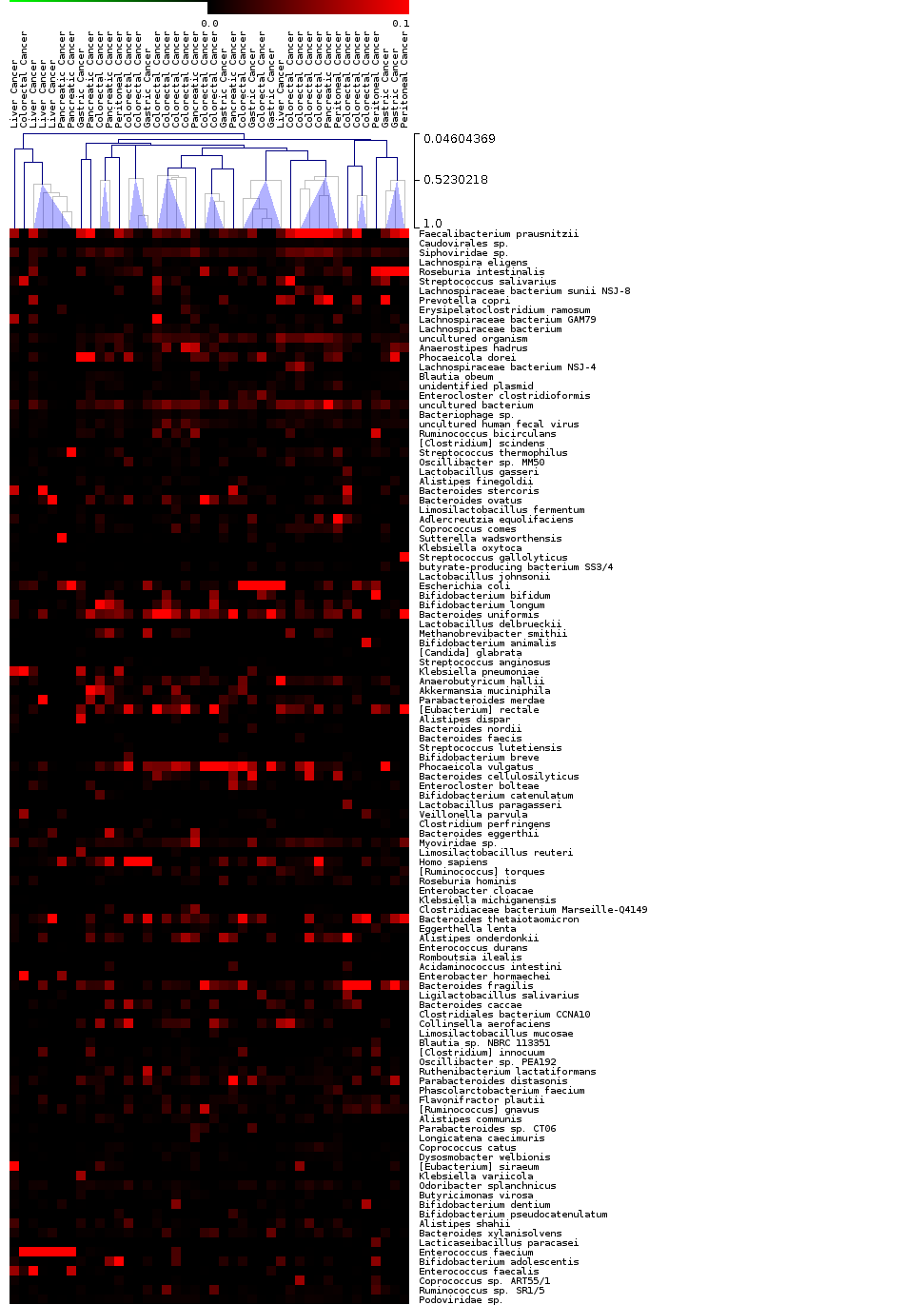

Supplement: Supplementary file 3 — Figure S1C: Supplementary Information. [file JCSM-17-e70324-s001.tiff]

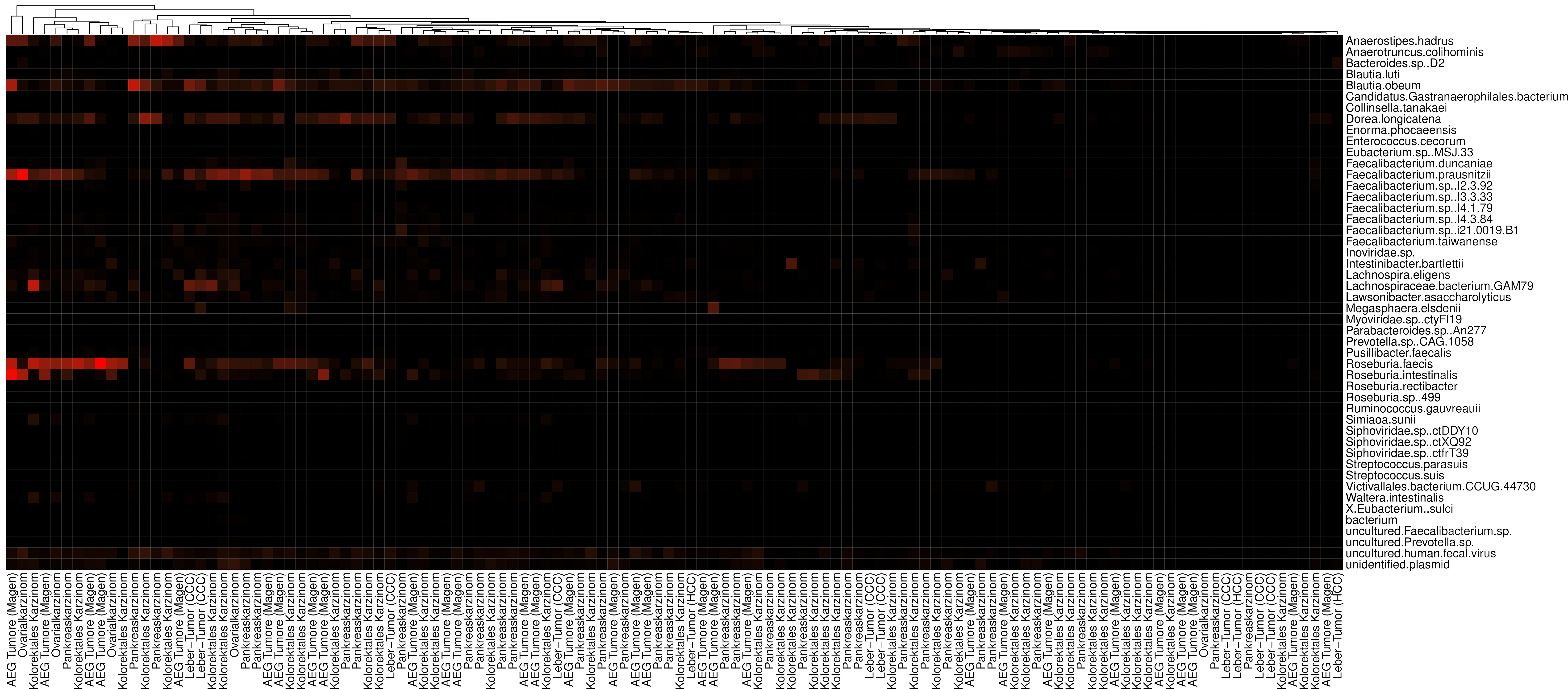

Supplement: Supplementary file 4 — Figure S1D: Supplementary Information. [file JCSM-17-e70324-s013.tiff]

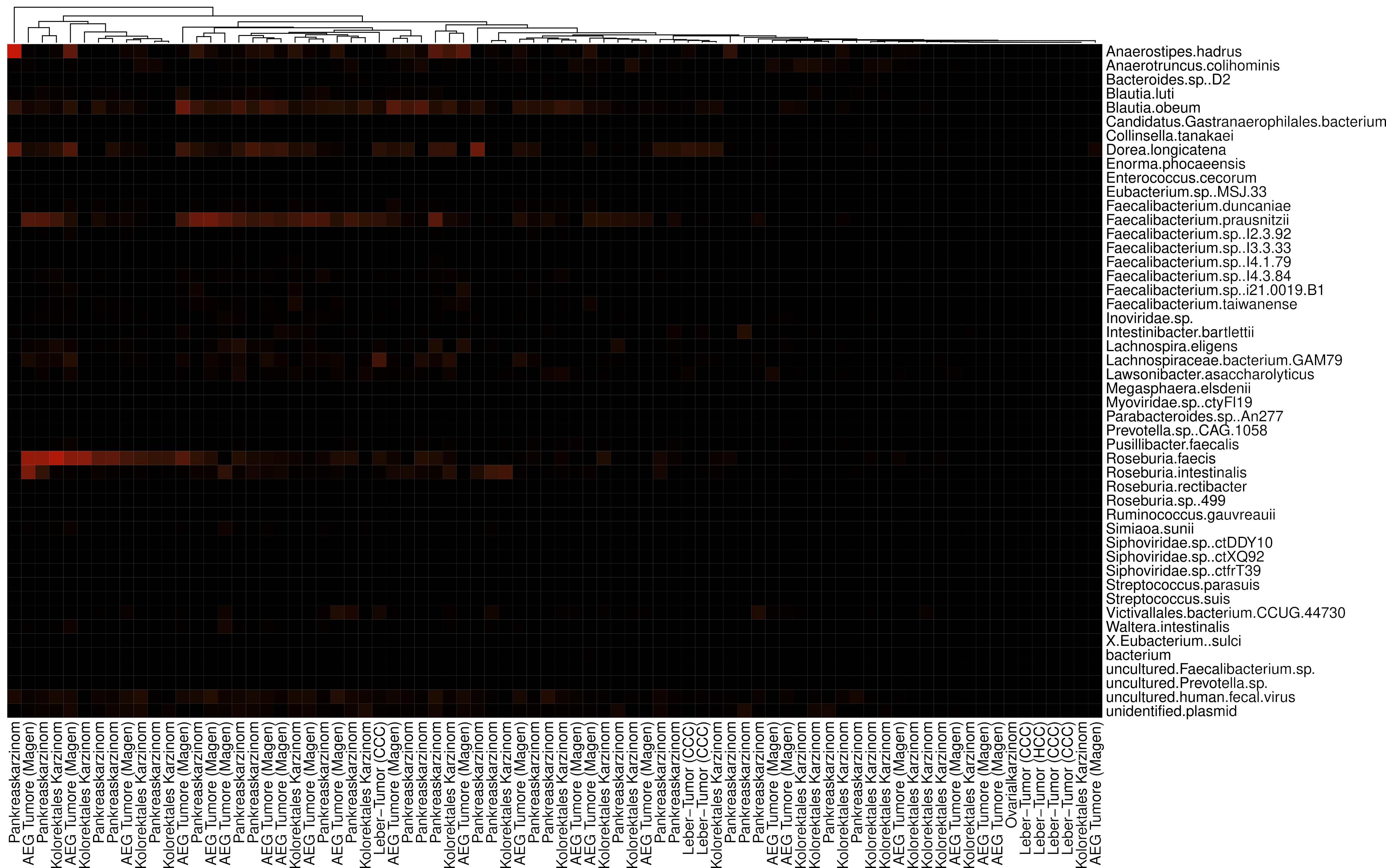

Supplement: Supplementary file 5 — Figure S1E: Supplementary Information. [file JCSM-17-e70324-s010.tiff]

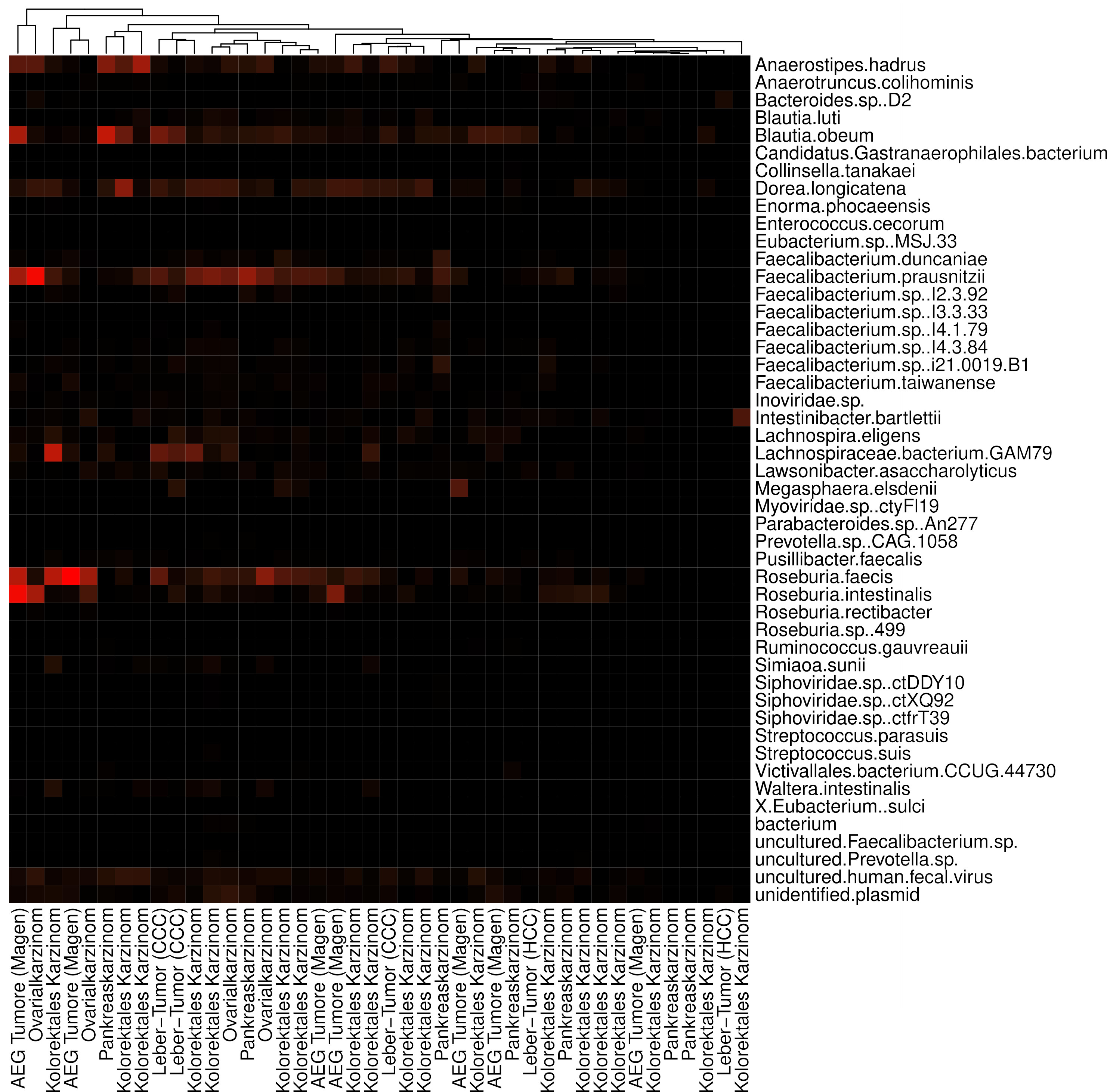

Supplement: Supplementary file 6 — Figure S1F: Supplementary Information. [file JCSM-17-e70324-s016.tiff]
