# Supplementary material for: Lowered Abundance of Gut Bacteriophage Species Is Associated With Human Cancer Cachexia
Source: J Cachexia Sarcopenia Muscle. 2026 Jun 7;17(3):e70324. doi: 10.1002/jcsm.70324 (PMC13243887; doi:10.1002/jcsm.70324)
Supplement: Supplementary file 7 — Table S1A: Species derived from 1.312 taxa inferred by read‐based mapping with Kraken/Bracken2 k‐mer matching against the NT‐database in 2022 under the morphology‐based ICTV phage taxonomy that showed overlapping significance for differential abundance by FDR‐corrected p values of < 0.05 in both two‐tailed unpaired t‐test (pairwise comparison of non‐transformed mean abundance values) and LinDA analysis (linear regression of log2‐transformed data) between cachectic (n = 78) compared to non‐cachectic cancer patients (n = 42). Species abundance is represented as compositional data, expressed as relative mean abundance (dimensionless proportion of the total metagenomics dataset) for each taxon, and transformed using the centered log‐ratio transformation before linear regression was applied. [file JCSM-17-e70324-s018.docx]

| **Supplementary Table S1A.** Species derived from 1.312 taxa inferred by read-based mapping with Kraken/Bracken2 *k-mer* matching against the NT-database in 2022 under the morphology-based ICTV phage taxonomy that showed overlapping significance for differential abundance by FDR-corrected P values of < 0.05 in both two-tailed unpaired *t*-test (pairwise comparison of non-transformed mean abundance values) and LinDA analysis (linear regression of log2-transformed data) between cachectic (n = 78) compared to non-cachectic cancer patients (n = 42). Species abundance is represented as compositional data, expressed as relative mean abundance (dimensionless proportion of the total metagenomics dataset) for each taxon, and transformed using the centered log-ratio transformation before linear regression was applied. | | | | |
| --- | --- | --- | --- | --- |
| Species | Mean basic abundance | Log2-fold change | FDR-corrected  P value  LinDA analysis | FDR-corrected  P value  *t*-test |
| Caudovirales sp. | 0.000284 | -0.6303 | 0.0390 | 0.0024 |
| Faecalibacterium prausnitzii | 0.003071 | -1.7236 | 0.0224 | 0.0019 |
| Lachnospira eligens | 0.000175 | -2.0826 | 0.0013 | 0.0052 |
| Lachnospiraceae bacterium | 0.000252 | -1.8396 | 0.0014 | 0.0452 |
| Lachnospiraceae bacterium sunii NSJ-8 | 0.000537 | -1.5400 | 0.0074 | 0.0314 |
| Roseburia intestinalis | 0.001592 | -1.4379 | 0.0137 | 0.0242 |
| Siphoviridae sp. | 0.005757 | -0.5568 | 0.0021 | 0.0037 |
| Streptococcus salivarius | 0.004653 | -1.4821 | 0.0318 | 0.0281 |
