# Supplementary material for: Lowered Abundance of Gut Bacteriophage Species Is Associated With Human Cancer Cachexia
Source: J Cachexia Sarcopenia Muscle. 2026 Jun 7;17(3):e70324. doi: 10.1002/jcsm.70324 (PMC13243887; doi:10.1002/jcsm.70324)
Supplement: Supplementary file 9 — Table S2A: Species derived from 1.312 taxa inferred by read‐based mapping with Kraken/Bracken2 k‐mer matching against the NT‐database in 2022 under the morphology‐based ICTV phage taxonomy that showed nominal significance for differential abundance (p < 0.05) in two‐tailed unpaired t‐test (pairwise comparison of non‐transformed mean abundance values) between cachectic (n = 78) compared to non‐cachectic cancer patients (n = 42). Species abundance is expressed as relative mean abundance (dimensionless proportion of the total metagenomics dataset) for each taxon. [file JCSM-17-e70324-s015.docx]

| **Supplementary Table S2A.** Species derived from 1.312 taxa inferred by read-based mapping with Kraken/Bracken2 k-mer matching against the NT-database in 2022 under the morphology-based ICTV phage taxonomy that showed nominal significance for differential abundance (P < 0.05 in two-tailed unpaired t-test (pairwise comparison of non-transformed mean abundance values) between cachectic (n = 78) compared to non-cachectic cancer patients (n = 42). Species abundance is expressed as relative mean abundance (dimensionless proportion of the total metagenomics dataset) for each taxon. | | | | |
| --- | --- | --- | --- | --- |
| Species | Mean basic abundance Whole cohort  (n = 120) | Mean basic abundance  Cachexia  (n = 78) | Mean basic abundance Non-cachexia  (n = 42) | P value *t*-test |
| Inoviridae sp. | 0.0007 | 0.0005 | 0.0010 | 0.0014 |
| Oscillibacter sp. MM59 | 0.0004 | 0.0003 | 0.0006 | 0.0138 |
| Caudovirales sp. ctt3K6 | 0.0001 | 0.0000 | 0.0001 | 0.0151 |
| Caudovirales sp. ct0YK8 | 0.0001 | 0.0000 | 0.0001 | 0.0199 |
| Microviridae sp. | 0.0001 | 0.0000 | 0.0001 | 0.0208 |
| Siphoviridae sp. ct9mC1 | 0.0001 | 0.0000 | 0.0001 | 0.0275 |
| Prevotella intermedia | 0.0001 | 0.0000 | 0.0002 | 0.0293 |
| Megasphaera elsdenii | 0.0007 | 0.0000 | 0.0021 | 0.0310 |
| Enterococcus lactis | 0.0003 | 0.0001 | 0.0008 | 0.0314 |
| Lachnospiraceae bacterium sunii | 0.0022 | 0.0012 | 0.0042 | 0.0314 |
| Prevotella copri | 0.0086 | 0.0051 | 0.0153 | 0.0370 |
| Faecalibacillus intestinalis | 0.0002 | 0.0001 | 0.0004 | 0.0378 |
| Siphoviridae sp. ctA995 | 0.0001 | 0.0000 | 0.0001 | 0.0407 |
| Erysipelatoclostridium ramosum | 0.0013 | 0.0008 | 0.0021 | 0.0408 |
| Lachnospiraceae bacterium KGMB03038 | 0.0002 | 0.0001 | 0.0003 | 0.0415 |
| Myoviridae sp. ctSGr1 | 0.0001 | 0.0000 | 0.0001 | 0.0432 |
| Lachnospiraceae bacterium GAM79 | 0.0043 | 0.0025 | 0.0076 | 0.0439 |
| Siphoviridae sp. ctZi05 | 0.0001 | 0.0000 | 0.0001 | 0.0464 |
| Longibaculum sp. KGMB06250 | 0.0002 | 0.0001 | 0.0003 | 0.0464 |
| Siphoviridae sp. ctX8T1 | 0.0001 | 0.0000 | 0.0001 | 0.0469 |
| Myoviridae sp. ctQf419 | 0.0001 | 0.0000 | 0.0001 | 0.0477 |
