# Supplementary material for: Lowered Abundance of Gut Bacteriophage Species Is Associated With Human Cancer Cachexia
Source: J Cachexia Sarcopenia Muscle. 2026 Jun 7;17(3):e70324. doi: 10.1002/jcsm.70324 (PMC13243887; doi:10.1002/jcsm.70324)
Supplement: Supplementary file 10 — Table S2B: Species derived from 1.513 taxa inferred by read‐based mapping with Kraken/Bracken2 k‐mer matching against the NT‐database in 2022 under the morphology‐based ICTV phage taxonomy that showed nominal significance for differential abundance (p < 0.05) in two‐tailed unpaired t‐test (pairwise comparison of non‐transformed mean abundance values) between cachectic (n = 78) compared to non‐cachectic cancer patients (n = 42). Species abundance is expressed as relative mean abundance (dimensionless proportion of the total metagenomics dataset) for each taxon. [file JCSM-17-e70324-s019.docx]

| **Supplementary Table S2B.** Species derived from 1.513 taxa inferred by read-based mapping with Kraken/Bracken2 *k*-mer matching against the NT-database in 2025 under the revised genome-based ICTV phage taxonomy that showed nominal significance for differential abundance (P < 0.05 in two-tailed unpaired t-test) between cachectic (n = 78) compared to non-cachectic cancer patients (n = 42). Species abundance is expressed as relative mean abundance (dimensionless proportion of the total metagenomics dataset) for each taxon. | | | | |
| --- | --- | --- | --- | --- |
| Species | Mean basic abundance Whole cohort  (n = 120) | Mean basic abundance  Cachexia  (n = 78) | Mean basic abundance Non-cachexia (n = 42) | P value  t-test |
| uncultured human fecal virus | 0.003400 | 0.002640 | 0.004812 | 0.004076 |
| Faecalibacterium prausnitzii | 0.012149 | 0.008802 | 0.018364 | 0.004136 |
| Lachnospira eligens | 0.001418 | 0.000891 | 0.002396 | 0.005008 |
| Inoviridae sp. | 0.000387 | 0.000299 | 0.000550 | 0.005426 |
| Faecalibacterium sp. I3-3-33 | 0.000013 | 0.000007 | 0.000023 | 0.006315 |
| Anaerotruncus colihominis | 0.000805 | 0.001071 | 0.000311 | 0.007566 |
| Lachnospiraceae bacterium GAM79 | 0.003451 | 0.001653 | 0.006791 | 0.008592 |
| Siphoviridae sp. ctXQ92 | 0.000027 | 0.000014 | 0.000051 | 0.009409 |
| Pusillibacter faecalis | 0.000297 | 0.000217 | 0.000445 | 0.009450 |
| Eubacterium sp. MSJ-33 | 0.000011 | 0.000005 | 0.000022 | 0.015173 |
| Enorma phocaeensis | 0.000023 | 0.000009 | 0.000049 | 0.015247 |
| Siphoviridae sp. ctDDY10 | 0.000016 | 0.000007 | 0.000035 | 0.015307 |
| Roseburia rectibacter | 0.000065 | 0.000042 | 0.000109 | 0.015619 |
| Waltera intestinalis | 0.000465 | 0.000246 | 0.000871 | 0.016487 |
| Siphoviridae sp. ctZi05 | 0.000001 | 0.000000 | 0.000001 | 0.016649 |
| Parabacteroides sp. An277 | 0.000006 | 0.000001 | 0.000014 | 0.018770 |
| uncultured Faecalibacterium sp. | 0.000001 | 0.000001 | 0.000003 | 0.019509 |
| Lawsonibacter asaccharolyticus | 0.000898 | 0.000679 | 0.001304 | 0.020021 |
| Roseburia intestinalis | 0.005036 | 0.002930 | 0.008948 | 0.021340 |
| [Eubacterium] sulci | 0.000002 | 0.000001 | 0.000005 | 0.021673 |
| Faecalibacterium sp. I4-3-84 | 0.000423 | 0.000320 | 0.000614 | 0.021745 |
| Candidatus Gastranaerophilales bacterium | 0.000001 | 0.000000 | 0.000003 | 0.021891 |
| Blautia obeum | 0.009767 | 0.007629 | 0.013735 | 0.021914 |
| Prevotella sp. CAG:1058 | 0.000020 | 0.000008 | 0.000041 | 0.025303 |
| Megasphaera elsdenii | 0.000531 | 0.000001 | 0.001515 | 0.027095 |
| Myoviridae sp. ctyFl19 | 0.000002 | 0.000001 | 0.000004 | 0.028595 |
| Blautia luti | 0.000708 | 0.000525 | 0.001046 | 0.028660 |
| Faecalibacterium sp. I4-1-79 | 0.000131 | 0.000072 | 0.000242 | 0.032124 |
| uncultured Prevotella sp. | 0.000032 | 0.000011 | 0.000071 | 0.032699 |
| Simiaoa sunii | 0.000372 | 0.000187 | 0.000715 | 0.032755 |
| Lachnospiraceae bacterium oral taxon 096 | 0.000001 | 0.000000 | 0.000002 | 0.035750 |
| Myoviridae sp. ctSGr1 | 0.000001 | 0.000000 | 0.000002 | 0.036543 |
| Roseburia faecis | 0.011638 | 0.008753 | 0.016996 | 0.037520 |
| Roseburia sp. 499 | 0.000001 | 0.000000 | 0.000001 | 0.037567 |
| Collinsella tanakaei | 0.000006 | 0.000001 | 0.000014 | 0.038404 |
| Dorea longicatena | 0.008196 | 0.006663 | 0.011041 | 0.039506 |
| Enterococcus cecorum | 0.000003 | 0.000002 | 0.000005 | 0.040227 |
| Victivallales bacterium CCUG 44730 | 0.000496 | 0.000705 | 0.000108 | 0.040814 |
| Streptococcus parasuis | 0.000010 | 0.000005 | 0.000019 | 0.041404 |
| Bacteroides sp. D2 | 0.000188 | 0.000080 | 0.000390 | 0.042082 |
| Siphoviridae sp. ctfrT39 | 0.000005 | 0.000001 | 0.000012 | 0.042742 |
| bacterium | 0.000082 | 0.000062 | 0.000120 | 0.045529 |
| Anaerostipes hadrus | 0.006906 | 0.005071 | 0.010314 | 0.049249 |
| Streptococcus suis | 0.000018 | 0.000007 | 0.000039 | 0.049281 |
| Weissella confusa | 0.000003 | 0.000000 | 0.000008 | 0.049542 |
