# Supplementary material for: Lowered Abundance of Gut Bacteriophage Species Is Associated With Human Cancer Cachexia
Source: J Cachexia Sarcopenia Muscle. 2026 Jun 7;17(3):e70324. doi: 10.1002/jcsm.70324 (PMC13243887; doi:10.1002/jcsm.70324)
Supplement: Supplementary file 21 — Table S11A: Classifier importance (≥ 0.30) in random forest‐based machine learning models running under metagenomics data of taxa inferred by read‐based mapping with k‐mer matching from the NT‐database in 2022 under the morphology‐based phage taxonomy as input features for classification between cachectic (n = 78) and non‐cachectic cancer patients (n = 42). Classifier importance was assessed using the metrics mean decrease in impurity and the number of nodes in which the predictor appeared (Gini importance). Cancer patients (n = 42). [file JCSM-17-e70324-s005.docx]

| **Supplementary Table S11A.** Classifier importance (≥ 0.30) in random forest-based machine learning models running under metagenomics data of taxa inferred by read-based mapping with *k-mer* matching from the NT-database in 2022 and under the morphology-based ICTV phage taxonomy as input features for classification between cachectic (n = 78) and non-cachectic cancer patients (n = 42). Classifier importance was assessed using the metrics mean decrease in impurity and the number of nodes in which the predictor appeared (Gini importance). | |
| --- | --- |
| Classifier importance | Species name |
| 0.49 | Siphoviridae sp. |
| 0.46 | Faecalibacterium prausnitzii |
| 0.43 | Streptococcus thermophilus |
| 0.43 | [Eubacterium] rectale |
| 0.43 | Roseburia intestinalis |
| 0.42 | Streptococcus salivarius |
| 0.40 | Prevotella copri |
| 0.38 | Anaerostipes hadrus |
| 0.37 | Ruminococcus gnavus |
| 0.37 | unidentified plasmid |
| 0.35 | uncultured human fecal virus |
| 0.34 | Ruminococcus sp. SR1/5 |
| 0.33 | Lachnospiraceae bacterium GAM79 |
| 0.33 | Alistipes dispar |
| 0.32 | Lactobacillus gasseri |
| 0.32 | Lachnospiraceae bacterium sunii NSJ-8 |
| 0.31 | Coprococcus sp. ART55/1 |
| 0.31 | Erysipelatoclostridium ramosum |
| 0.30 | Caudovirales sp. |
