# Supplementary material for: Lowered Abundance of Gut Bacteriophage Species Is Associated With Human Cancer Cachexia
Source: J Cachexia Sarcopenia Muscle. 2026 Jun 7;17(3):e70324. doi: 10.1002/jcsm.70324 (PMC13243887; doi:10.1002/jcsm.70324)
Supplement: Supplementary file 22 — Table S11B: Classifier importance (≥ 0.30) in random forest‐based machine learning models running under metagenomics data of taxa inferred by read‐based mapping with k‐mer matching from the NT‐database in 2025 under the revised genome‐based ICTV phage taxonomy as input features for classification between cachectic (n = 78) and non‐cachectic cancer patients (n = 42). Classifier importance was assessed using the metrics mean decrease in impurity and the number of nodes in which the predictor appeared (Gini importance). [file JCSM-17-e70324-s022.docx]

| **Supplementary Table S11B.** Classifier importance (≥ 0.30) in random forest-based machine learning models running under metagenomics data of taxa inferred by read-based mapping with *k-mer* matching from the NT-database in 2025 under the revised genome-based ICTV phage taxonomy as input features for classification between cachectic (n = 78) and non-cachectic cancer patients (n = 42). Classifier importance was assessed using the metrics mean decrease in impurity and the number of nodes in which the predictor appeared (Gini importance). | |
| --- | --- |
| Classifier importance | Species name |
| 0.43 | Candidatus Gastranaerophilales bacterium |
| 0.40 | Myoviridae sp. ctyFl19 |
| 0.39 | Streptococcus thermophilusFaecalibacterium sp. I4-1-79 |
| 0.39 | Blautia luti |
| 0.38 | Lachnospira eligens |
| 0.38 | Siphoviridae sp. ctDDY10 |
| 0.38 | Bacteroides sp. D2 |
| 0.37 | Faecalibacterium sp. I3-3-33 |
| 0.37 | Faecalibacterium duncaniae |
| 0.36 | Anaerostipes hadrus |
| 0.35 | Dorea longicatena |
| 0.35 | Faecalibacterium sp. I4-3-84 |
| 0.34 | Anaerotruncus colihominis |
| 0.34 | Roseburia intestinalis |
| 0.33 | Streptococcus suis |
| 0.33 | Faecalibacterium sp. I2-3-92 |
| 0.33 | Inoviridae sp. |
| 0.33 | Faecalibacterium taiwanense |
| 0.32 | Faecalibacterium prausnitzii |
| 0.32 | Parabacteroides sp. An277 |
| 0.32 | unidentified plasmid |
| 0.31 | Blautia obeum |
| 0.31 | Prevotella sp. CAG:1058 |
| 0.3 | Simiaoa sunii |
| 0.3 | Roseburia rectibacter |
| 0.3 | Faecalibacterium sp. i21-0019-B1 |
