# Supplementary material for: Lowered Abundance of Gut Bacteriophage Species Is Associated With Human Cancer Cachexia
Source: J Cachexia Sarcopenia Muscle. 2026 Jun 7;17(3):e70324. doi: 10.1002/jcsm.70324 (PMC13243887; doi:10.1002/jcsm.70324)
Supplement: Supplementary file 24 — Table S12B: Performance and classification metrics of random forest‐based machine learning models running under the metagenomics data inferred from the NT‐database in 2025 and the revised genome‐based ICTV phage taxonomy for classification between cachectic (n = 78) and non‐cachectic cancer patients (n = 42). Performance parameters shown are based on 10‐fold cross‐validation and a meta‐classifier approach to make base classifier cost‐sensitive. [file JCSM-17-e70324-s006.docx]

| **Supplementary Table S12B.** Performance and classification metrics of random forest-based machine learning models running run under the metagenomics data inferred from the NT-database in 2025 and the revised genome-based ICTV phage taxonomy for classification between cachectic (n = 78) and non-cachectic cancer patients (n = 42). Performance parameters shown are based on 10-fold cross-validation and a meta-classifier approach to make base classifier cost-sensitive. | | |
| --- | --- | --- |
| Database approach | NT-  database | *De novo* Assembled  Viral Contigs |
| Figure | 5A | 5B |
| Dataset | Whole cohort | Whole cohort |
| Microbiome samples, (n) | 120 | 120 |
| True positive, (n) | 64 | 66 |
| True negative, (n) | 22 | 24 |
| False positive, (n) | 20 | 18 |
| False negative, (n) | 14 | 12 |
| Accuracy (correctly classified) | 0.716 | 0.75 |
| Area under ROC | 0.727 | 0.703 |
| Precision | 0.709 | 0.744 |
| Recall | 0.717 | 0.750 |
| F1-measure | 0.711 | 0.754 |
