# Supplementary material for: Lowered Abundance of Gut Bacteriophage Species Is Associated With Human Cancer Cachexia
Source: J Cachexia Sarcopenia Muscle. 2026 Jun 7;17(3):e70324. doi: 10.1002/jcsm.70324 (PMC13243887; doi:10.1002/jcsm.70324)
Supplement: Supplementary file 25 — Data S1: Supplementary Information. [file JCSM-17-e70324-s020.docx]

Supporting References

1. Koh K, Scott R, Cespedes Feliciano EM, Janowitz T, Goncalves MD, White EP, et al. Cancer-associated cachexia: bridging clinical findings with mechanistic insights in human studies. *Cancer Discov.* 2025;15:1543-1568.
2. Roeland EJ, Bohlke K, Baracos VE, Bruera E, Del Fabbro E, Dixon S, et al. Management of Cancer Cachexia: ASCO Guideline. *J Clin Oncol*. 2020;38:2438-2453.
3. Godsil M, Ritz NL, Venkatesh S, Meeske AJ. Gut phages and their interactions with bacterial and mammalian hosts. *J Bacteriol.* 2025;207:e0042824.
4. Yu X, Cheng L, Yi X, Li B, Li X, Liu X, et al. Gut phageome: challenges in research and impact on human microbiota. *Front Microbiol.* 2024;15:1379382.
5. Shkoporov AN, Turkington CJ, Hill C. Mutualistic interplay between bacteriophages and bacteria in the human gut. *Nat Rev Microbiol*. 2022;20:737-749.
6. Tiamani K, Luo S, Schulz S, Xue J, Costa R, Khan Mirzaei M, Deng L. The role of virome in the gastrointestinal tract and beyond. *FEMS Microbiol Rev.* 2022;46:fuac027.
7. Bikel S, López-Leal G, Cornejo-Granados F, Gallardo-Becerra L, García-López R, Sánchez F, et al. Gut dsDNA virome shows diversity and richness alterations associated with childhood obesity and metabolic syndrome. *iScience.* 2021;24:102900.
8. Yang K, Niu J, Zuo T, Sun Y, Xu Z, Tang W, et al. Alterations in the gut virome in obesity and type 2 diabetes mellitus. Gastroenterology 2021;161:1257-1269.e13
9. Fan G, Cao F, Kuang T, Yi H, Zhao C, Wang L, et al. Alterations in the gut virome are associated with type 2 diabetes and diabetic nephropathy. Gut Microbes. 2023;15:2226925.
10. Mirzaei MK, Khan AA, Ghosh P, Taranu ZE, Taguer M, Ru J, et al. Bacteriophages isolated from stunted children can regulate gut bacterial communities in an age-specific manner. *Cell Host Microbe* 2020;27:199-212.
11. Visconti A, Le Roy CI, Rosa F, Rossi N, Martin TC, Mohney RP, et al. Interplay between the human gut microbiome and host metabolism. *Nat Commun.* 2019;10(1):4505.
12. Nguyen S, Baker K, Padman BS, Patwa R, Dunstan RA, Weston TA, et al. Bacteriophage transcytosis provides a mechanism to cross epithelial cell layers. *mBio.* 2017;8(6):e01874-17.
13. Van Belleghem JD, Dąbrowska K, Vaneechoutte M, Barr JJ, Bollyky PL. Interactions between bacteriophage, bacteria, and the mammalian immune system. *Viruses.* 2018;11(1):10.
14. Chica Cardenas LA, Leonard MM, Baldridge MT, Handley SA. Gut virome dynamics: from commensal to critical player in health and disease. *Nat Rev Gastroenterol Hepatol.* 2026;23(2):126-144.
15. Xia Y, Sun J. Hypothesis testing and statistical analysis of microbiome. *Genes Dis*. 2017;4:138-148
16. Ma C, Bandukwala S, Burman D, Bryson J, Seccareccia D, Banerjee S, et al. Interconversion of three measures of performance status: an empirical analysis. *Eur J Cancer*. 2010;46:3175-3183.
17. Chen X, Liu X, Ji W, Zhao Y, He Y, Liu Y, et al. The PG-SGA outperforms the NRS 2002 for nutritional risk screening in cancer patients: a retrospective study from China. *Front Nutr.* 2023;10:1272420.
18. Hui D, Bruera E. The Edmonton Symptom Assessment System 25 years later: past, present, and future developments. *J Pain Symptom Manage.* 2017;53:630-643.
19. Higgins-Biddle JC, Barbor TF. A review on the alcohol use disorders identification test (AUDIT), AUDIT-C, and USAUDIT for screening in the United States: past issues and future directions. *Am J Drug Alcohol Abuse*. 2018;44:578-586.
20. Bridgers J, Alexander K, Karsan A. Operationalizing quality assurance for clinical Illumina somatic next-generation sequencing pipelines. *J Mol Diagn.* 2024;26(2):96-105.
21. Lu J, Breitwieser FP, Thielen P, Salzberg SL. Bracken: estimating species abundance in metagenomics data. *Peer J Comput Sci.* 2017;3:e104.
22. Li J, Jia H, Cai X, Zhong H, Feng Q, Sunagawa S, et al. An integrated catalogue of reference genes in the human gut microbiome. *Nat Biotechnol*. 2014;32:834-841.
23. Martí JM, Kok CR, Thissen JB, Mulakken NJ, Avila-Herrera A, Jaing CJ, et al. Addressing the dynamic nature of reference data: a new nucleotide database for robust metagenomic classification. *mSystems.* 2025;10(4):e0123924.
24. Gao S, Sun R, Singh R, Yu So S, Chan CTY, Savidge T, Hu M. The role of gut microbial beta-glucuronidase in drug disposition and development. *Drug Discov Today*. 2022;27:103316.
25. Kumar S, Lekshmi M, Parvathi A, Ojha M, Wenzel N, Varela MF. Functional and structural roles of the major facilitator superfamily bacterial multidrug efflux pumps. *Microorganisms.* 2020;8:266.
26. Kumawat M, Nabi B, Daswani M, Viquar I, Pal N, Sharma P, et al. Role of bacterial efflux pump proteins in antibiotic resistance across microbial species. *Microb Pathog.* 2023;181:106182.
27. Yutin N, Benler S, Shmakov SA, Wolf YI, Tolstoy I, Rayko M, et al. Analysis of metagenome-assembled viral genomes from the human gut reveals diverse putative CrAss-like phages with unique genome features. *Nat Commun* 2021;12:1044.
28. Edwards RA, Vega AA, Norman HM, Ohaeri M, Levi K, Dinsdale EA, et al. Global phylogeography and ancient evolution of the widespread human gut virus crAssphage. *Nat Microbiol.* 2019;4:1727-1736.
29. Shkoporov AN, Clooney AG, Sutton TDS, Ryan FJ, Daly KM, Nolan JA, et al. The human gut virome is highly diverse, stable, and individual specific. *Cell Host Microbe* 2019;26:527-541.
30. Gulyaeva A, Garmaeva S, Ruigrok RAAA, Wang D, Riksen NP, Netea MG, et al. Discovery, diversity, and functional associations of crAss-like phages in human gut metagenomes from four Dutch cohorts. *Cell Rep.* 2022;38:110204.
31. Schmidtke DT, Hickey AS, Wirbel J, Lin JD, Liachko I, Sherlock G, et al. The prototypic crAssphage is a linear phage-plasmid. *Cell Host Microbe.* 2025;33:1347-1362.e5.
32. Tomofuji Y, Kishikawa T, Maeda Y, Ogawa K, Nii T, Okuno T, et al. Whole gut virome analysis of 476 Japanese revealed a link between phage and autoimmune disease. *Ann Rheum Dis.* 2022;81:278-288.
33. Cervantes-Echeverría M, Gallardo-Becerra L, Cornejo-Granados F, Ochoa-Leyva A. The Two-Faced Role of crAssphage Subfamilies in Obesity and Metabolic Syndrome: Between Good and Evil. *Genes (Basel).* 2023;14:139.
34. Clooney AG, Sutton TDS, Shkoporov AN, Holohan RK, Daly KM, O´Regan O, et al. Whole-virome analysis sheds light on viral dark matter in inflammatory bowel disease. *Cell Host Microbe* 2019;26:764-778.
35. Mirzaei MK, Maurice CF. Ménage á trois in the human gut: interactions between host, bacteria and phages. *Nat Rev Microbiol*. 2017;15:397-408.
36. Tobin CA, Hill C, Shkoporov AN. Factors affecting variation of the human gut phageome. *Ann Rev Microbiol* 2023;77:363-379.
37. Dahlman S, Avellaneda-Franco L, Rutten EL, Gulliver EL, Solari S, Chonwerawong M, et al. Isolation, engineering and ecology of temperate phages from the human gut. *Nature.* 2025;647:698-705.
38. Zhang S, Easwaran M, Elafify M, Mahmoud AA, Wang X, Ahn J. Prophages and their interactions with lytic phages in the human gut microbiota and their impact on microbial diversity, gut health, and disease. *Appl Environ Microbiol.* 2025;91(12):e0189925.
39. Cervantes-Echeverría M, Jimenez-Rico MA, Manzo R, Hernández-Reyna A, Cornejo-Granados F, Bikel S, et al. A. Human-derived fecal virome transplantation (FVT) reshapes the murine gut microbiota and virome, enhancing glucose regulation. *PLoS One.* 2025;20:e0337760.
40. Mao X, Larsen SB, Zachariassen LSF, Brunse A, Adamberg S, Mejia JLC, et al. Transfer of modified gut viromes improves symptoms associated with metabolic syndrome in obese male mice. *Nat Commun.* 2024;15:4704.
41. Ritz NL, Draper LA, Bastiaanssen TFS, Turkington CJR, Peterson VL, van de Wouw M, et al. The gut virome is associated with stress-induced changes in behaviour and immune responses in mice. *Nat Microbiol.* 2024;9:359-376.
42. Zuo T, Lu XJ, Zhang Y, Cheung CP, Lam S, Zhang F, et al. Gut mucosal virome alterations in ulcerative colitis. *Gut.* 2019;68:1169-1179.
43. Gogokhia L, Buhrke K, Bell R, Hoffman B, Brown DG, Hanke-Gogokhia C, et al. Expansion of bacteriophages is linked to aggravated intestinal inflammation and colitis. *Cell Host Microbe.* 2019;25:285-299.
44. Sinha A, Li Y, Mirzaei MK, Shamash M, Samadfam R, King IL, et al. Transplantation of bacteriophages from ulcerative colitis patients shifts the gut bacteriome and exacerbates the severity of DSS colitis. *Microbiome*. 2022;10:105.
45. Yadegar A, Bar-Yoseph H, Monaghan TM, Pakpour S, Severino A, Kuijper EJ, et al. Fecal microbiota transplantation: current challenges and future landscapes. *Clin Microbiol Rev*. 2024;37:e0006022.
46. Sutcliffe SG, Shamash M, Hynes AP, Maurice CF. Common oral medications lead to prophage induction in bacterial isolates from the human gut. *Viruses* 2021;13:445.
47. Wang Y, Zhang Y, Lane NE, Wu J, Yang T, Li J, et al. Population-based metagenomics analysis reveals altered gut microbiome in sarcopenia: data from the Xiangya Sarcopenia Study. *J Cachexia Sarcopenia Muscle.* 2022;13:2340-2351.
48. Smith SE, Huang W, Tiamani K, Unterer M, Mirzaei KM, Deng L. Emerging technologies in the study of the virome. *Curr Opin Virol*. 2022;54:101231.
